# Effect of Medication Reconciliation at Hospital Admission on 30-Day Returns to Hospital: A Randomized Clinical Trial

**DOI:** 10.1001/jamanetworkopen.2021.24672

**Published:** 2021-09-16

**Authors:** Alessandro Ceschi, Roberta Noseda, Michela Pironi, Nicole Lazzeri, Ottavia Eberhardt-Gianella, Saida Imelli, Sara Ghidossi, Stefano Bruni, Alberto Pagnamenta, Paolo Ferrari

**Affiliations:** 1 Division of Clinical Pharmacology and Toxicology, Institute of Pharmacological Sciences of Southern Switzerland, Ente Ospedaliero Cantonale, Lugano, Switzerland; 2 Clinical Trial Unit, Ente Ospedaliero Cantonale, Lugano, Switzerland; 3 Faculty of Biomedical Sciences, Università della Svizzera Italiana, Lugano, Switzerland; 4 Department of Clinical Pharmacology and Toxicology, University Hospital Zurich, Zurich, Switzerland; 5 Hospital Pharmacy Service, Institute of Pharmacological Sciences of Southern Switzerland, Ente Ospedaliero Cantonale, Lugano, Switzerland; 6 Department of Information and Communications Technology, Ente Ospedaliero Cantonale, Bellinzona, Switzerland; 7 Department of Intensive Care, Ente Ospedaliero Cantonale, Bellinzona, Switzerland; 8 Division of Pneumology, University of Geneva, Geneva, Switzerland; 9 Department of Nephrology, Ente Ospedaliero Cantonale, Lugano, Switzerland; 10 Prince of Wales Hospital Clinical School, University of New South Wales, Sydney, New South Wales, Australia

## Abstract

**Importance:**

According to international recommendations, hospitals should use medication reconciliation to prevent medication errors and improve patient safety.

**Objective:**

To assess the impact of medication reconciliation at hospital admission on patient-centered health care outcomes.

**Design, Setting, and Participants:**

This parallel group, open-label randomized controlled trial used centralized randomization to the intervention group (ie, individuals with medication reconciliation) or control group (ie, individuals with only standard, physician-acquired medication history). Outcome assessors and data analysts were blinded to group allocation. Participants included 1702 patients aged 85 years or older, with more than 10 medications at hospital admission, or meeting both conditions at 2 regional secondary teaching hospitals in southern Switzerland. Study duration was 14.5 months, from November 1, 2018, to January 15, 2020. Data were analyzed from December 2018 through March 2020.

**Interventions:**

Medication reconciliation was performed at hospital admission in 3 steps: (1) the pharmacy assistant obtained the list of the patient’s current medications (ie, the best possible medication history [BPMH]); (2) the clinical pharmacist led reconciliation of the BPMH with the list of home medications recorded at hospital admission by the attending physician (according to the hospital standard procedure); and (3) medication discrepancies were communicated to the attending physician, and, when necessary, medications prescribed at admission were adapted.

**Main Outcomes and Measures:**

The primary outcome was a composite postdischarge health care use variable quantified as the proportion of patients with unplanned all-cause hospital visits (including visits to the emergency department and hospital readmissions) within 30 days after discharge from the hospital when medication reconciliation took place. A time-to-event analysis was performed.

**Results:**

Among 1702 patients (median [interquartile range] age, 86.0 [79.0-89.0] years; 720 [42.3%] men), 866 patients (50.9%) were allocated to the intervention group and 836 patients (49.1%) to the control group. The primary outcome occurred among 340 participants (39.3%) in the intervention group and 330 participants (39.5%) in the control group (*P* = .93). In time-to-event analyses at study closeout, unplanned all-cause hospital visits to the emergency department (log-rank *P* = .08) and unplanned all-cause hospital readmissions (log-rank *P* = .10) occurred similarly in the intervention and control groups.

**Conclusions and Relevance:**

These findings suggest that medication reconciliation at hospital admission has no impact on postdischarge health care outcomes among patients aged 85 years or older, with more than 10 medications at hospital admission, or meeting both conditions.

**Trial Registration:**

ClinicalTrials.gov Identifier: NCT03654963

## Key Points

**Question:**

Is medication reconciliation effective in improving health care outcomes when targeted to patients at increased risk of medication discrepancies and medication-related harms because of older age, polypharmacy, or both conditions?

**Findings:**

In this randomized clinical trial of 1702 Swiss patients aged 85 years or older, with more than 10 medications at hospital admission, or meeting both conditions, the proportion of patients with unplanned all-cause hospital returns within 30 days after initial discharge did not differ among those who received medication reconciliation at hospital admission vs those who did not.

**Meaning:**

Despite the clinical validity of medication reconciliation, this study did not find an impact of this intervention on patient-centered health care outcomes among a selected patient population at increased risk of medication discrepancies.

## Teaser

This randomized clinical trial examines the effect of medication reconciliation on unplanned hospital returns among Swiss patients who were older, had polypharmacy, or met both conditions.

## Introduction

Medication reconciliation is the systematic process of obtaining the most accurate list of all medications a patient is currently taking (ie, the best possible medication history [BPMH]), including medication name, dosage, frequency, and route. This list is to be compared with the list of medications ordered by a physician at admission, transfer, or discharge in order to provide correct medications to the patient at all interfaces of care within the hospital.^1^

According to international recommendations, hospitals should use medication reconciliation to prevent medication errors and improve patient safety.^2,3,4^ In Switzerland, the Swiss Patient Safety Foundation, following the national pilot program “Progress! Safe Pharmacotherapy at the Interface Points” (2014-2016), published recommendations for safe pharmacotherapy at interfaces of care, emphasizing the importance of carrying out systematic medication reconciliation.^5^ In addition, the Swiss Federal Office of Public Health promotes the introduction of medication reconciliation in Swiss health care institutions.^6^

Errors in medication use process are among the most common causes of in-hospital morbidity and mortality.^7^ Unintended medication discrepancies (eg, omissions, duplications, and dosing errors) can occur across transitions of care and, if not identified and resolved, may place the patient at risk of medication-related harm, thus negatively impacting the quality and safety of patient care.^8^ Studies from 2012^9^and 2019^10^ suggest that medication reconciliation reduces the number of medication discrepancies. However, because of heterogeneity among studies; variation in type, intensity, and duration of interventions; and differences in timing of follow-up measurements in the most recent systematic reviews, meta-analyses, and overviews of systematic reviews, evidence for the effectiveness of medication reconciliation in patient-centered outcomes and health care use is inconclusive.^11,12^

To date, 1 randomized clinical trial (RCT), not yet included in recent systematic reviews or meta-analyses, found that an extended intervention composed of medication review at admission and medication reconciliation at discharge significantly reduced the number of emergency department (ED) visits and readmissions within 6 months from discharge compared with usual care at 4 acute admission wards in Denmark.^13^ Although the study was adequately powered, with a large sample size, the assessment of the primary composite outcome at 6 months could have been biased by factors unrelated to the targeted intervention on medication use. Indeed, medication-related readmissions within 6 months of inclusion were not significantly reduced in the extended intervention group compared with the usual care group. Moreover, the study population included patients aged 18 years or older with 5 or more medications at hospital admission. Therefore, older patients with fewer than 5 medications were not included in the study. However, these patients represent a subpopulation with increased frailty and increased risk of adverse health outcomes. For these patients, medication use (regardless of the overall number of medications taken) can be complex and potentially associated with medication-related problems and increased morbidity and mortality, likely imposing a large economic burden on health care resources.^14,15,16^

Considering polypharmacy (ie, having >10 medications at hospital admission), older patient age (ie, ≥85 years), or both conditions as inclusion criteria, this parallel group RCT aimed to assess the impact of medication reconciliation at hospital admission on a composite postdischarge health care use variable. This was quantified as the proportion of patients with unplanned all-cause hospital visits (including visits to the ED and hospital readmissions) within 30 days after initial discharge, a time when patients may still be using the medications they were using at discharge and a reasonable latency period between intervention and assessed outcomes.

## Methods

This RCT was approved by the local ethics committee of Swissethics, the Swiss Association of Research Ethics Committees, with exemption from obtaining informed consent for participants allocated to the control group following the Zelen design.^17^ A copy of the study protocol is available in Supplement 1. All patient data obtained from the control group were routinely recorded in electronic health records (EHRs), without need of obtaining additional data for the study. All participants randomized to the intervention group received a participant information sheet and a consent form describing the study and providing sufficient information to make an informed decision about their participation in the study. When recruited patients were cognitively unable to meaningfully participate in the study, the participant information sheet and consent form were provided to the individuals’ caregivers or legal representatives, who were afterward actively involved (on behalf of cognitively impaired patients) during pharmacist assistant–led interviews used obtain information for the BPMH. This study followed Consolidated Standards of Reporting Trials (CONSORT) reporting guideline.

### Study Design and Participants

This was a parallel group RCT conducted at 2 secondary teaching hospitals in southern Switzerland from November 1, 2018, to January 15, 2020. These hospitals are part of the Ente Ospedaliero Cantonale (EOC) network of public hospitals, which comprises 4 sites across southern Switzerland.

Eligible participants were patients aged 85 years or older, with more than 10 medications at hospital admission (ie, home medications recorded by the attending physician in an unstructured and nonsystematic manner), or meeting both conditions. Patients were excluded if they were admitted to an intensive care unit without reaching inpatient wards, had planned hospital stays shorter than 48 hours, or were admitted to an EOC hospital ward within the previous 3 months with discharge at home.

All consecutive eligible patients admitted to the hospitals were identified through the hospital EHRs and recruited if aged 85 years or older, if they had more than 10 medications at hospital admission, or if they met both conditions. Each participant was enrolled 1 time only over the study period.

The parallel group design included the intervention group, with medication reconciliation, and the control group, with standard, physician-acquired medication history. In the control group, recording of patient home medications occurred in an unstructured manner, with neither the option to consult pharmacists nor the possibility for the attending physician to directly access EHR data from other regional or national hospitals, clinics, or pharmacies to review medication lists and refill histories.

### Randomization and Blinding Procedures

**Figure 1.**
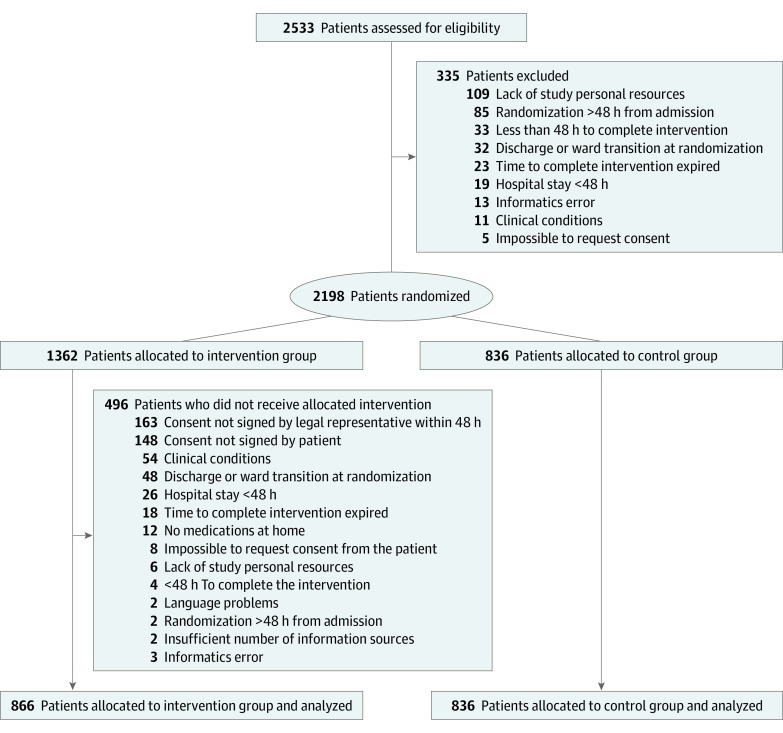
Study Flowchart

Simple randomization across study sites was carried out by the EOC clinical trial unit by creating a unique list of randomized patients using computerized random number generation. Allocation of randomized participants was initially set as 1:1. However, at 3 months from the start of the study, the number of participants allocated to the intervention and control groups was unbalanced because 496 of 1362 participants (36.4%) allocated to the intervention group had not received the intervention for several reasons (Figure 1). Therefore, randomization was recalibrated to a 2:1 allocation (intervention group vs control group).

The trial was open label. Therefore, owing to the nature of the intervention, clinical pharmacists (M.P., N.L., O.E.G., S.I., and S.G.) and pharmacy assistants, along with participants allocated to the intervention group, were aware of the study, while outcome assessors (R.N. and A.P.) and data analysts (R.N., S.B., and A.P.) remained blinded.

### Procedures

Eligible patients randomized to the intervention group received medication reconciliation according to 3 steps: At first, the pharmacy assistant compiled an ex novo comprehensive list of the patient’s current medications at hospital admission (including medications used in chronic therapy and those taken on an as-needed basis) and obtained the BPMH. To confirm the accuracy of the BPMH, the pharmacy assistant used 2 or more sources of information, including, when possible, an interview with the patient or the patient’s caregiver or legal representative, in addition to referral letters and prescription and medication lists from primary care physicians, community pharmacists, nursing homes, and home care. Pharmacy assistants received training and instructions from clinical pharmacists (M.P., N.L., O.E.G., S.I., and S.G.) face to face and through guiding documents to learn about medication reconciliation procedures and to familiarize themselves with the use of EHRs. This was done to enhance data quality and decrease the amount of missing or incomplete data, inaccuracies, and excessive variability in measurements. Clinical pharmacists had a consolidated experience in the medication reconciliation process from a previous in-house pilot study^18^ and previous working experience abroad, where the clinical pharmacist is responsible for the entire medication reconciliation process. In the subsequent step, the clinical pharmacist reconciled the BPMH with the list of home medications recorded at hospital admission by the attending physician (according to the hospital standard procedure). To resolve medication discrepancies that were unclear or difficult to evaluate and to propose adaptation of the pharmacotherapy prescribed at hospital admission, the clinical pharmacist referred to the attending physician. Lastly, physicians determined changes to patient pharmacotherapy. Throughout the intervention, the pharmacy assistant actively involved patients (or their caregivers or legal representatives) only during the interview. Medication reconciliation was performed within 48 hours from participant recruitment in the study. Patients allocated to the control group received only the standard physician-acquired medication history at hospital admission.

Baseline data collected from each participant consisted of age at admission, sex, number of medications at hospital admission, primary diagnosis, and disposition status. The following variables were additionally collected solely in the intervention group: number of medication discrepancies, number of unjustified medications added, number of omitted medications, number of incorrect medications by frequency, number of incorrect medications by name, number of incorrect dose strengths, number of incorrect galenic forms (ie, the pharmaceutical formulation of a medication), interview duration, time required to obtain BPMH, and overall duration of medication reconciliation procedure. These variables, along with numerical identification codes (defined by the EOC Information and Communications Technology Department [ICT]), first collected within a database created ad hoc for study purpose were subsequently transferred to the electronic case report form (CRF), for which the ICT (which was responsible for data management) and the biostatistician (A.P. and S.B.) had authorization for data entry.

### Outcomes

The primary outcome was a composite postdischarge health care use variable quantified as the proportion of patients with unplanned all-cause hospital visits (including visits to the ED and hospital readmissions, including to the intensive care unit, at any of 4 regional hospitals encompassed by the EOC) within 30 days after initial discharge. Planned visits, which do not involve going through the ED, were not counted in the assessment of the primary outcome. These data were retrieved by the ICT from existing administrative data sources and integrated within the CRF.

Secondary outcomes were assessed during the first inpatient stay (ie, when study recruitment occurred) and consisted of the period prevalence of adverse drug events (ADEs) occurring during the hospital stay, length of hospital stay (LOS), number of in-hospital deaths, and number of resources used during the hospital stay (ie, laboratory tests, radiologic exams, and electrocardiograms). The active pharmacovigilance system at the Regional Pharmacovigilance Centre of southern Switzerland was used to measure the prevalence of ADEs during the hospital stay. This is an electronic system that actively and continuously screens EHRs for predefined words and word combinations related to ADEs (eMethods in Supplement 2). Therefore, EHRs identified through this system and referring to patients aged 85 years or older, with more than 10 medications at admission, or meeting both conditions were manually assessed for a drug-event causal relationship (using the World Health Organization [WHO]–Uppsala Monitoring Centre causality assessment system^19^) and validated to discard false-positive cases. Subsequently, ADEs were categorized as nonserious or serious (using WHO seriousness criteria for resulting in death, being life threatening, prolonging hospitalization, resulting in persistent disability or incapacity, or determining other clinical conditions). The outcome assessor (R. N.) was masked to group allocation (without access to the database of baseline data or CRF) and had practical experience in causality assessment from daily pharmacovigilance activity. The number of in-hospital deaths, laboratory tests, radiographic exams, and electrocardiograms and the LOS were retrieved from administrative data sources that were already in use at the EOC for other purposes. The ICT integrated all secondary outcome measures within the CRF.

### Sample Size Estimation

Based on previous data extracted from the EOC administrative databases, a positive composite outcome variable was expected in approximately 30% of participants from the control group. To detect a minimal clinically relevant difference (set at 6%) in that composite outcome variable between the intervention and the control groups with a 0.80 statistical power and a .05 significance level, a sample size of 1718 patients was estimated by Pearson χ^2^ test. Sample size calculation was performed using PASS statistical software version 15.0.3 (NCSS).

### Statistical Analysis

An independent biostatistician (A.P.) blind to trial group allocation analyzed the data following a prespecified statistical analysis plan. Quantitative data were summarized as median with interquartile range (IQR). Qualitative data were presented as absolute numbers with percentages. For the primary outcome, the proportion of patients with unplanned all-cause hospital visits (including visits to the ED and hospital readmissions) within 30 days after initial discharge was compared in the intervention vs the control group using the χ^2^ or Fisher exact test, as appropriate.

A time-to-event analysis was performed at study closeout (ie, 30 days after inclusion of the last patient). This was done to assess differences in the occurrence of unplanned all-cause hospital visits to the ED and unplanned all-cause readmissions to ward in the intervention vs the control groups.

Outcome variables were unplanned all-cause hospital visits to the ED and unplanned all-cause hospital readmissions, separately assessed by Kaplan-Meier curves. Time-to-event curves in the intervention and control groups were compared using the log-rank test. All tests were performed 2-sided, and *P* < .05 was considered statistically significant. All statistical analyses were performed using Stata statistical software version 15 (StataCorp). Data were analyzed from December 2018 through March 2020.

## Results

Among 2533 patients eligible for the study, 1702 individuals were included. Median (IQR) patient age was 86.0 (79.0-89.0) years, and 720 (42.3%) were men. There were 866 participants (50.9%) assigned to the intervention group and 836 participants (49.1%) assigned to the control group (Figure 1). The median (IQR) age was 86.0 (79.0-89.0) years for the intervention group and 86.0 (79.8-90.0) years for the control group; there were 500 (57.7%) women in the intervention group and 482 (57.7%) women in the control group. Table 1 shows the baseline characteristics of study participants.

**Table 1.** Baseline Characteristics of Study Participants

Characteristic	No. (%)	
	Intervention group (n = 866)	Control group (n = 836)
Age ≥85, y	314 (36.3)	397 (47.5)
>10 medications at hospital admission	301 (34.8)	295 (35.3)
Age ≥85 y and >10 medications at hospital admission	251 (29.0)	144 (17.2)
Age, median (IQR), y	86.0 (79.0-89.0)	86.0 (79.8-90.0)
Men	366 (42.3)	354 (42.3)
Women	500 (57.7)	482 (57.7)
No. of medications at hospital admission, median (IQR)	12 (9-16)	11 (6-13)
Primary diagnosis^a^		
Certain infectious and parasitic diseases	26 (3.0)	27 (3.2)
Diseases of the blood and blood-forming organs and certain disorders involving the immune mechanism	7 (0.8)	10 (1.2)
Diseases of the circulatory system	138 (15.9)	148 (17.7)
Diseases of the digestive system	88 (10.2)	93 (11.1)
Diseases of the ear and mastoid process	8 (0.9)	5 (0.6)
Diseases of the eye and adnexa	1 (0.1)	0
Diseases of the genitourinary system	74 (8.5)	49 (5.9)
Diseases of the musculoskeletal system and connective tissue	73 (8.4)	59 (7.1)
Diseases of the nervous system	31 (3.6)	25 (3.0)
Diseases of the respiratory system	105 (12.1)	102 (12.2)
Diseases of the skin and subcutaneous tissue	9 (1.0)	16 (1.9)
Endocrine, nutritional, and metabolic diseases	19 (2.2)	24 (2.9)
Factors influencing health status and contact with health services	0	2 (0.2)
Injury, poisoning, and certain other consequences of external causes	142 (16.4)	132 (15.8)
Mental and behavioral disorders	48 (5.5)	40 (4.8)
Neoplasms	45 (5.2)	43 (5.1)
Symptoms, signs, and abnormal clinical and laboratory findings not elsewhere classified	52 (6.0)	61 (7.3)
Disposition status		
Home	545 (62.9)	478 (57.2)
Nonmedical institution or nursing home	221 (25.5)	229 (27.4)
Non-medical institution	3 (0.3)	6 (0.7)
Rehabilitation institution	46 (5.3)	52 (6.2)
Hospital or clinic	19 (2.2)	29 (3.5)
Psychiatric institution or clinic	13 (1.5)	19 (2.3)
Death	19 (2.2)	23 (2.8)

Abbreviation: IQR, interquartile range.

^a^ Diagnosis categories are from the *International Statistical Classification of Diseases and Related Health Problems, Tenth Revision* (*ICD-10*).

Among 866 patients from the intervention group, 830 individuals (96.0%) had 1 or more medication discrepancies, with a median (IQR) 6 (4-9) discrepancies per patient. Among these participants, 797 individuals (96.0%) had medication discrepancies in chronic therapy (median [IQR] 4 [3-7] discrepancies per patient receiving chronic therapy). The most frequently occurring discrepancies among patients in the intervention group were medication omission (567 participants [68.3%] from the intervention group had medication omissions in chronic therapy, and 517 participants [62.3%] had medication omissions in as-needed therapy), followed by incorrect medication name, incorrect dosage regime, incorrect dose amount, unjustified medications not prescribed to the patient at home, and incorrect galenic forms (Table 2).

**Table 2.** Medication Discrepancies in the Intervention Group

Discrepancy type	Discrepancies per patient, median (IQR)	Patients with discrepancies, No. (%) (N = 830)
Medication discrepancies		
Chronic treatment	4 (3-7)	797 (96.0)
As needed	2 (1-4)	581 (70.0)
Omission		
Chronic treatment	2 (1-4)	567 (68.3)
As needed	2 (1-3)	517 (62.3)
Incorrect medication name		
Chronic treatment	2 (1-3)	435 (52.4)
As needed	1 (1-1)	13 (1.6)
Incorrect dosage regime		
Chronic treatment	1 (1-2)	335 (40.4)
As needed	1 (1-1.5)	75 (9.0)
Incorrect dose amount		
Chronic treatment	1 (1-2)	350 (42.2)
As needed	1 (1-1)	43 (5.2)
Unjustified medications (not prescribed to the patient at home)		
Chronic treatment	1 (1-2)	228 (27.5)
As needed	1 (1-2)	55 (6.6)
Incorrect galenic forms		
Chronic treatment	1 (1-1)	88 (10.6)
As needed	1 (1-1)	11 (1.3)

Abbreviation: IQR, interquartile range.

The median (IQR) time for a complete intervention of medication reconciliation was 55 (40-70) minutes. This included a median (IQR) 15 (10-25) minutes for the interview and 40 (30-50) minutes for the search, analysis, and validation of the sources of information used to obtain the BPMH; evaluation of medication discrepancies; and communication of discrepancies to the attending physician.

### Primary Outcome

**Figure 2.**
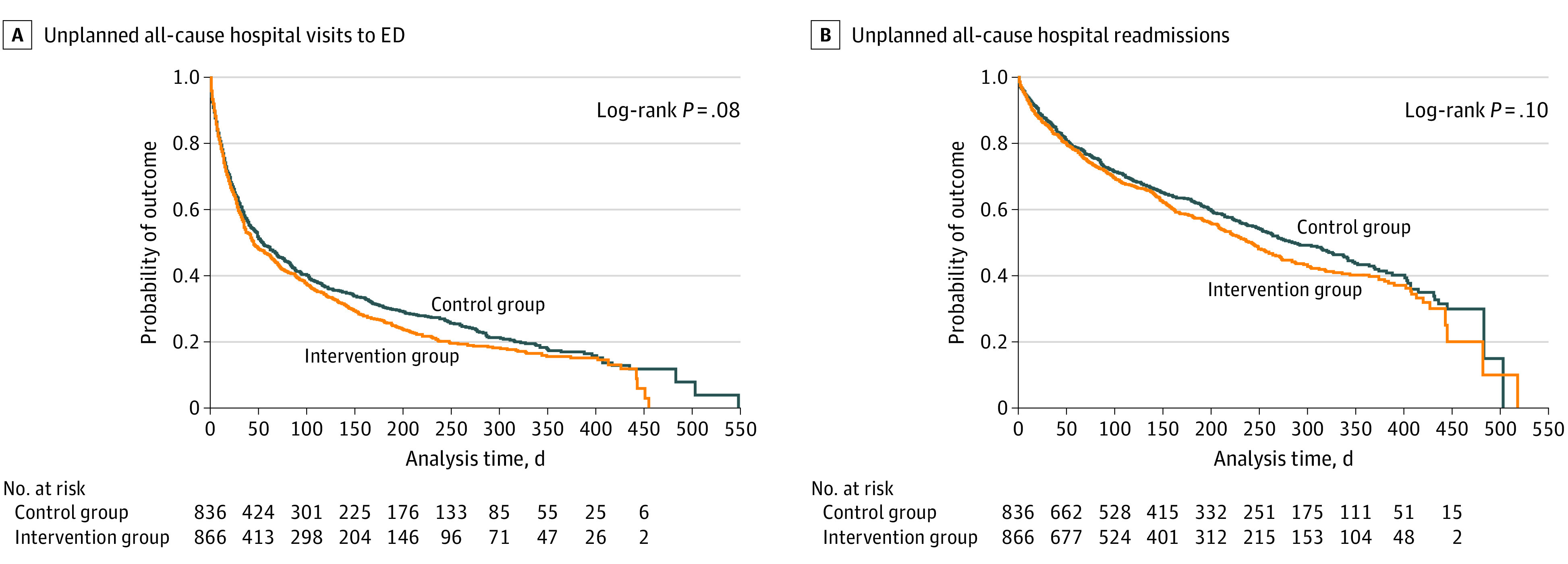
Kaplan-Meier Survival Estimates for Outcomes Since Study-Related Discharge

The primary outcome occurred among 340 participants (39.3%) in the intervention group and 330 participants (39.5%) in the control group (*P* = .93). In time-to-event analyses at study closeout, unplanned all-cause hospital visits to the ED occurred similarly in the intervention and control groups (*P* = .08) (Figure 2A). No statistically significant difference was found for unplanned all-cause hospital readmissions in the intervention group vs control group (*P* = .10) (Figure 2B).

### Secondary Outcomes

The overall period prevalence of ADEs (including serious and not serious ADEs) occurring during the hospital stay was 11 ADEs (1.3%) in the intervention group and 14 ADEs (1.7%) in the control group (*P* = .49). When only serious ADEs that prolonged hospitalization were evaluated, no statistically significant difference was found between the intervention group (5 ADEs [0.6%]) and control group (6 ADEs [0.7%]; *P* = .77). Median (IQR) LOS was 8 (5-13) days in the intervention group and 8 (4-13) days in the control group (*P* = .23). There were 19 in-hospital deaths (2.2%) in the intervention group and 23 such deaths (2.8%) in the control group (*P* = .55). The median (IQR) number of laboratory tests was 9.5 (4.0-23.0) in the intervention group and 9.0 (4.0-20.0) in the control group (*P* = .31). The 2 groups each had a median (IQR) 1 (0-2) radiologic exams and 1 (0-1) electrocardiograms.

## Discussion

In this RCT, medication reconciliation performed at hospital admission in a selected patient population aged 85 years or older, with more than 10 medications at hospital admission, or meeting both conditions had no impact on the proportion of patients with unplanned all-cause hospital visits (ie, ED visits and hospital readmissions) within 30 days after initial discharge. Moreover, unplanned all-cause hospital visits to the ED and unplanned all-cause hospital readmissions during the study period occurred similarly in the intervention and control groups. No effect was found on secondary health care outcomes, defined as the prevalence of ADEs during the hospital stay, LOS, in-hospital deaths, and resources used during the hospital stay quantified as number of laboratory tests, radiologic exams, and electrocardiographic exams.

The results of the present study are in line with the current uncertain and low-quality evidence regarding the measurable effect of medication reconciliation on patient-centered clinical outcomes.^10,11,12^ Although there is ample evidence of the effect of medication reconciliation in reducing the number of medication discrepancies,^20^ the evidence on the clinical impact of such a reduction is equivocal because of heterogeneity in settings, patient populations, and typologies of interventions across studies.^10,11,12^

To date, 1 RCT^13^ has found that an extended intervention composed of medication review at admission and medication reconciliation at discharge significantly reduced the number of ED visits and readmissions within 6 months from discharge compared with usual care at 4 acute admission wards in Denmark. Nevertheless, the assessment of the primary composite outcome at 6 months in that RCT could have been biased by factors unrelated to the targeted intervention on medication use, as confirmed when medication-related readmissions within 6 months of inclusion were assessed and no significant reduction was found in the extended intervention group compared with the usual care group. Aiming at selecting a patient population that could have reasonably benefited from medication reconciliation at hospital admission, restricted inclusion criteria based on older age (ie, aged ≥85 years), polypharmacy (arbitrarily defined as >10 medications at admission), or both conditions were applied in our trial. While polypharmacy is often defined as routinely taking a minimum of 5 medications,^21^ there is no standard, universally accepted definition.^14^ Indeed, assigning a numeric threshold is not always useful, especially when considering older patients with age-related physiological changes, a greater degree of frailty, and multiple coexisting conditions.^22^ Regardless of the number of medications used, older patients represent a population at increased risk of drug-drug interactions, ADEs, falls, cognitive impairment, and nonadherence.^15,23,24^ On the other hand, although the prevalence of polypharmacy increases as the population ages and experiences multiple chronic conditions,^14^ polypharmacy also concerns other age groups, including children and younger adults with specific diagnoses.^25^ Moreover, regardless of age, as the number of medications increases, the rate of medication discrepancies increases.^18^ Consistently, the patient population in the present study also included younger adults with more than 10 medications at hospital admission.

Most patients who received medication reconciliation had 1 or more medication discrepancies, while, for ethical reasons, it was not possible to assess medication discrepancies in the control group. Assuming a similar risk of medication discrepancies at baseline in the intervention and control groups, patient harm from medication discrepancies, quantified as the proportion of unplanned all-cause hospital visits (ie, ED visits and hospital readmissions) occurring at the latest within 30 days from discharge was similar in the 2 groups.

Concerning secondary health care outcomes evaluated in this study, medication reconciliation did not show any effect. In the intervention and control groups, the number of ADEs that occurred during hospital stays was relatively low, similar to the prevalence reported in inpatient settings in other countries.^26^ Moreover, previous studies^11^ assessing the impact of medication reconciliation on ADEs did not find differences as compared to usual care. The system used in our study to detect ADEs was based on a reliable methodology of active pharmacovigilance previously set up at the Regional Pharmacovigilance Centre of southern Switzerland at the EOC and calibrated to minimize the false positive rate.^27^ Notably, such a system assumed that key terms referring to possible ADEs were recorded in patients’ EHRs by health care personnel, a requisite that could have potentially been met by a minority of personnel only, thus potentially resulting in a low ADE rate at baseline.

A 2021 prepost study^28^ involving patients with colorectal cancer and other chronic diseases undergoing elective colorectal surgery did not find a statistically significant change in mean LOS following a multifaceted program of medication reconciliation at all transitions of care. Mortality as clinical outcome for all causes or related to medication has been assessed by few studies at various time points, and they did not find a significant reduction in mortality after medication reconciliation.^11,12,29^ Additionally, resource use quantified as numbers of laboratory tests, radiologic exams, and electrocardiographic exams resulted in low numbers in our study that did not allow any reasonable conclusion.

### Strengths and Limitations

This trial has several strengths that challenge the quality of the literature that claims evidence of medication reconciliation effectiveness on patient-centered clinical outcomes.^10,11,12^ Inclusion criteria were defined in order to select a patient population reasonably associated with an increased risk of medication errors and potentially related harms. Because of complex health conditions associated with older age irrespective of the number of medications at admission or the high number medications at admission taken by younger patients, the study population represented a suitable target population among which medication reconciliation may have produced measurable effects on clinical outcomes. Pharmacy assistants and clinical pharmacists led the intervention according to a well-established, structured stepwise procedure, which was easily implemented. In addition, the study has several methodological strengths, including independent observers for outcome verification and the largest, to our knowledge, randomly selected patient sample size to date.

This study also has several limitations that may explain why we did not find an effect of medication reconciliation at hospital admission on direct patient outcomes. First, the primary composite outcome was assessed regardless of the causes that led to a hospital revisit within 30 days from discharge. This choice could have masked the real proportion of hospital revisits that occurred because of medication-related harms. Although hospital revisits that occurred because of medication-related harms were likely rare, they could have reasonably happened within 30 days from discharge, when patients were likely still using the medications they were using at discharge. Owing to the limited human resources available, medication discrepancies identified in the intervention group were not assessed for their clinical relevance in potentially causing an adverse drug event. Such an assessment could have provided useful information about the potential risk of hospital returns because of medication-related harms at 30 days from discharge or later. In this context, in addition to the assessment of hospital returns, it could have been worth exploring out-of-hospital visits in the ambulatory care setting. Second, the choice of performing a single-component intervention of medication reconciliation at admission was a double edge-sword. On the one hand, it may have involved the most delicate phase among the interfaces of care. However, on the other hand, not having a continuum at the subsequent interfaces of care, especially not performing medication reconciliation at discharge, made finding a difference in the hard patient-centered outcomes very unlikely. Third, the design of the study, with the intervention performed at 2 hospitals within a regional subarea of Switzerland, reduced the generalizability of the findings. Indeed, at 30 days from discharge, a relatively high proportion of unplanned all-cause hospital visits (including ED visits and hospital readmissions) was observed in the control group. Although that high proportion could have been related to the multiple coexisting conditions that typically affect older patients (who represented most of the study population), lower proportions were reported in other centers in different countries.^13^ Moreover, the older age of study participants may have not adequately represented all patients for whom medication reconciliation may be indicated. A fourth potential problem was that of spillover or contamination, which could have occurred if physicians exposed to the intervention changed their practice by applying elements learned from the intervention to patients allocated to the control group. Fifth, another possible explanation for the lack of an effect found for medication reconciliation at hospital admission on direct patient outcomes may be that discrepancies identified during the intervention, albeit communicated by the clinical pharmacist to physicians, may not have been considered.

## Conclusions

The findings of this study suggesting that medication reconciliation at hospital admission had no impact on postdischarge health care outcomes among patients aged 85 years or older, with more than 10 medications at hospital admission, or meeting both conditions fit neatly into the ongoing debate about whether it is reasonable to expect an impact of medication reconciliation on health care outcomes.^30,31^ Although it is unlikely that medication reconciliation is ineffective, the overall quality of the literature remains mixed and low.^10,11,12^ By contrast, the face validity (ie, the clinical importance) of medication reconciliation is unquestionable, given that WHO and many other international organizations consider it a milestone in the control of safe medication use and a necessary part of good clinical care.^2,3,4^ Future studies are warranted to assess the overall impact of medication reconciliation at admission and discharge in the hospital, as well as in the ambulatory care setting, particularly among populations of any age with complex medication regimens.

## Notes

Supplement 1.
Trial Protocol

Supplement 2.
eMethods

Supplement 3.
Data Sharing Statement
