# Supplementary material for: Effect of Medication Reconciliation at Hospital Admission on 30-Day Returns to Hospital: A Randomized Clinical Trial
Source: JAMA Netw Open. 2021 Sep 16;4(9):e2124672. doi: 10.1001/jamanetworkopen.2021.24672 (PMC8446815; doi:10.1001/jamanetworkopen.2021.24672)
Supplement: Supplement 1. — Trial Protocol [file jamanetwopen-e2124672-s001.pdf]

Clinical Study Protocol

Parallel group randomized controlled trial to assess the impact of medication reconciliation at hospital admission on healthcare outcomes.

Short title: Medication reconciliation at hospital admission

Trial acronym: MedRec

Study Type:	Clinical trial with the therapeutic activity of medication reconciliation
Study Categorisation:	Risk category A
Study Registration:	Trial registered at *clinicaltrials.gov* (NCT03654963), and in the SNCTP on *kofam.ch* (via BASEC, ID 2018-01536)
Study Identifier:	n.a.
Sponsor, Sponsor-Investigator or Principal Investigator:	Sponsor:<br>Ente Ospedaliero Cantonale<br>Viale Officina 3<br>6501 Bellinzona - Switzerland<br>Email: direzione.generale@eoc.ch<br>Telephone: +41 91 8111301<br><br>Principal Investigator:<br>Prof. Dr. med. Alessandro Ceschi<br>Medical and scientific director<br>Istituto di Scienze Farmacologiche della Svizzera Italiana<br>Clinica di Farmacologia e Tossicologia Clinica<br>Ente Ospedaliero Cantonale<br>Via Tesserete 46<br>6903 Lugano – Switzerland<br>Email: alessandro.ceschi@eoc.ch<br>Telephone: +41 91 8116863
Investigational Product:	n.a.
Protocol Version and Date:	Version 5 (31/12/2019)

CONFIDENTIAL

The information contained in this document is confidential and property of the Direzione Generale Ente Cantonale Ospedaliero (EOC) in collaboration with the Istituto di Scienze Farmacologiche della Svizzera Italiana (ISFSI). The information may not - in full or in part - be transmitted, reproduced, published, or disclosed to others than the applicable Competent Ethics Committee (CEC), without prior written authorisation from the Sponsor and the Principal Investigator.

Signature Page(s)

Study number	Trial registered at *clinicaltrials.gov* (NCT03654963), and in the SNCTP on *kofam.ch* (via BASEC, ID 2018-01536)
Study Title	Parallel group randomized controlled trial to assess the impact of medication reconciliation at hospital admission on healthcare outcomes

The Principal Investigator, the co-investigator, the trial statistician, the scientific collaborator, the clinical pharmacist and the computer scientist, have approved the protocol version 1 (17.08.2018), and confirm hereby to conduct the study according to the protocol, current version of the World Medical Association Declaration of Helsinki, ICH-GCP guidelines or ISO 14155 norm if applicable and the local legally applicable requirements.

Principal Investigator:
Prof. Dr. med. Alessandro Ceschi

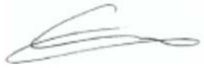

Lugano 20/08/2018

Signature

Co-Investigator
Prof. Dr. med. Paolo Ferrari

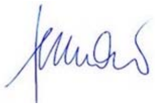

Bellinzona 20/08/2018

Signature

Trial statistician:
PD Dr. med. Alberto Pagnamenta

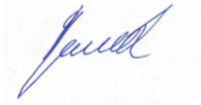

Mendrisio 20/08/2018

Signature

Scientific collaborator:
Roberta Noseda, PhD

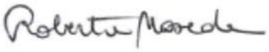

Lugano 20/08/2018

Signature

Clinical pharmacist:
Michela Pironi, MSc

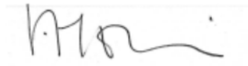

Locarno 20/08/2018

Signature

Computer scientist:
Stefano Bruni, BSc

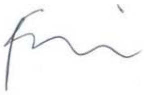

Bellinzona 20/08/2018

Signature

Local Principal Investigator at study site*:

I have read and understood this trial protocol and agree to conduct the trial as set out in this study protocol, the current version of the World Medical Association Declaration of Helsinki, ICH-GCP guidelines or ISO 14155 norm and the local legally applicable requirements.

Sites	Regional hospitals of Beata Vergine in Mendrisio and La Carità in Locarno
Principal investigator	Prof. Dr. med. Alessandro Ceschi (also Local Principal Investigator for both the two study sites)

Lugano 20/08/2018	
Place/Date	Signature

Note: In multicentre studies, this page must be individually signed by all participating Local Principal Investigators.

Table of Contents

STUDY SYNOPSIS **7**
ABBREVIATIONS **13**
STUDY SCHEDULE **14**
1. STUDY ADMINISTRATIVE STRUCTURE **15**
1.1 Sponsor, Sponsor-Investigator 15
1.2 Principal Investigator(s) 15
1.3 Statistician ("Biostatistician") 15
1.4 Laboratory 15
1.5 Monitoring institution 15
1.6 Data Safety Monitoring Committee 15
1.7 Any other relevant Committee, Person, Organisation, Institution 15
2. ETHICAL AND REGULATORY ASPECTS **17**
2.1 Study registration 17
2.2 Categorisation of study 17
2.3 Competent Ethics Committee (CEC) 17
2.4 Competent Authorities (CA) 17
2.5 Ethical Conduct of the Study 17
2.6 Declaration of interest 17
2.7 Patient Information and Informed Consent 17
2.8 Participant privacy and confidentiality 18
2.9 Early termination of the study 18
2.10 Protocol amendments 18
3. BACKGROUND AND RATIONALE **19**
3.1 Background and Rationale 19
3.2 Investigational Product (treatment, device) and Indication 19
3.3 Preclinical Evidence 19
3.4 Clinical Evidence to Date 19
3.5 Dose Rationale / Medical Device: Rationale for the intended purpose in study (pre-market MD) 19
3.6 Explanation for choice of comparator (or placebo) 19
3.7 Risks / Benefits 20
3.8 Justification of choice of study population 20
4. STUDY OBJECTIVES **21**
4.1 Overall Objective 21
4.2 Primary Objective 21
4.3 Secondary Objectives 21
4.4 Safety Objectives 21
5. STUDY OUTCOMES **22**
5.1 Primary Outcome 22
5.2 Secondary Outcomes 22
5.3 Other Outcomes of Interest 22
5.4 Safety Outcomes 22
6. STUDY DESIGN **23**
6.1 General study design and justification of design 23
6.2 Methods of minimising bias 23
6.2.1 Randomisation 23

6.2.2 Blinding procedures 23
6.2.3 Other methods of minimising bias 23
6.3 Unblinding Procedures (Code break) 23
7. STUDY POPULATION 24
7.1 Eligibility criteria 24
7.2 Recruitment and screening 24
7.3 Assignment to study groups 24
7.4 Criteria for withdrawal / discontinuation of participants 24
8. STUDY INTERVENTION 25
8.1 Identity of Investigational Products (treatment / medical device) 25
8.1.1 Experimental Intervention (treatment / medical device) 25
8.1.2 Control Intervention (standard/routine/comparator treatment / medical device) 25
8.1.3 Packaging, Labelling and Supply (re-supply) 25
8.1.4 Storage Conditions 25
8.2 Administration of experimental and control interventions 25
8.2.1 Experimental Intervention 25
8.2.2 Control Intervention 25
8.3 Dose / Device modifications 25
8.4 Compliance with study intervention 25
8.5 Data Collection and Follow-up for withdrawn participants 26
8.6 Trial specific preventive measures 26
8.7 Concomitant Interventions (treatments) 26
8.8 Study Drug / Medical Device Accountability 26
8.9 Return or Destruction of Study Drug / Medical Device 26
9. STUDY ASSESSMENTS 27
9.1 Study flow chart(s) / table of study procedures and assessments 27
9.2 Assessments of outcomes 27
9.2.1 Assessment of primary outcome 27
9.2.2 Assessment of secondary outcomes 27
9.2.3 Assessment of other outcomes of interest 27
9.2.4 Assessment of safety outcomes 27
9.2.5 Assessments in participants who prematurely stop the study 28
9.3 Procedures at each visit 28
9.3.1 Split into subtitles by type of visit 28
9.3.2 Split into subtitles by type of visit 28
9.3.3 Split into subtitles by type of visit 28
10. SAFETY 29
10.1 Drug studies 29
10.1.1 Definition and assessment of (serious) adverse events and other safety related events 29
10.1.2 Reporting of serious adverse events (SAE) and other safety related events 29
10.1.3 Follow up of (Serious) Adverse Events 29
10.2 Medical Device Category C studies 30
10.2.1 Definition and Assessment of (Serious) Adverse Events and other safety related events 30
10.2.2 Reporting of (Serious) Adverse Events and other safety related events 30
10.2.3 Follow up of (Serious) Adverse Events 30
10.3 Medical Device Category A studies 30

10.3.1 Definition and Assessment of safety related events 30
10.3.2 Reporting of Safety related events 30
10.4 Assessment, notification and reporting on the use of radiation sources 30
11. STATISTICAL METHODS 31
11.1 Hypothesis 31
11.2 Determination of Sample Size 31
11.3 Statistical criteria of termination of trial 31
11.4 Planned Analyses 31
11.4.1 Datasets to be analysed, analysis populations 31
11.4.2 Primary Analysis 31
11.4.3 Secondary Analyses 31
11.4.4 Interim analyses 32
11.4.5 Safety analysis 32
11.4.6 Deviation(s) from the original statistical plan 32
11.5 Handling of missing data and drop-outs 32
12. QUALITY ASSURANCE AND CONTROL 33
12.1 Data handling and record keeping / archiving 33
12.1.1 Case Report Forms 33
12.1.2 Specification of source documents 33
12.1.3 Record keeping / archiving 34
12.2 Data management 34
12.2.1 Data Management System 34
12.2.2 Data security, access and back-up 34
12.2.3 Analysis and archiving 34
12.2.4 Electronic and central data validation 34
12.3 Monitoring 34
12.4 Audits and Inspections 34
12.5 Confidentiality, Data Protection 34
12.6 Storage of biological material and related health data 34
13. PUBLICATION AND DISSEMINATION POLICY 35
14. FUNDING AND SUPPORT 36
14.1 Funding 36
14.2 Other Support 36
15. INSURANCE 36
16. REFERENCES 37
17. APPENDICES 38

190 **STUDY SYNOPSIS**

**Sponsor / Sponsor-Investigator**	Ente Ospedaliero Cantonale
**Study Title:**	Parallel group randomized controlled trial to assess the impact of medication reconciliation at hospital admission on healthcare outcomes.
**Short Title / Study ID:**	Medication reconciliation at hospital admission
**Protocol Version and Date:**	Version 4 (27/09/2019)
**Trial registration:**	Trial registered at *clinicaltrials.gov* (NCT03654963), and in the SNCTP on *kofam.ch* (via BASEC, ID 2018-01536).
**Study category and Rationale**	Study category A: low/absent potential risk for patients exposed to the intervention.<br>Information concerning patient medication history, gathered during medication reconciliation, will not imply any practical change on patients. This information will be used to correct potential omissions or other types of errors regarding the medication history originally obtained at admission. Medical doctors, not involved in the present study (to whom clinical pharmacists will communicate any type of discrepancy highlighted by medication reconciliation), will be in charge of deciding any change in drug therapy, directly interface with the patients and let them know about any changes in their pharmacotherapy.
**Clinical Phase:**	n.a.
**Background and Rationale:**	Background: Medication reconciliation is a systematic process by which health care professionals obtain the most complete and accurate information about the drugs and other products regularly taken by patients (**14**). Internationally, the value of this procedure is mainly attributed to the reduction in the number of adverse drug events (ADEs), which can cause drug-related morbidity and mortality, as well as unnecessary health care costs (**15-17**). The WHO indicates medication reconciliation as a priority strategy for patient safety (**14**).<br>Rationale: As part of the *Progress! Pilot project Safe Pharmacotherapy at the interface points* (**18**), promoted by the Federal Office of Public Health, coordinated by the Swiss Patients Safety Foundation and held in several Swiss hospitals, medication reconciliation at hospital admission was introduced at the regional hospital Beata Vergine in Mendrisio, from 2014 to 2016. During this pilot project it was shown that medication reconciliation after obtaining the "Best Possible Medication History" (BPMH) by a pharmacist at hospital admission, in comparison with the standard medication history obtained by the physician at admission, reduced the number of clinically relevant drug discrepancies: among 100 patients included in the project, an average of 5.23 discrepancies per patient was highlighted, and 47% of all patients had a clinically relevant discrepancy potentially evolving in a serious ADE (**19**).<br>Need for a trial: A structured, well-established and practicable procedure of medication reconciliation that improves patient safety assuring a better quality of care at hospital admission might provide evidence that medication reconciliation could be a valuable intervention to be applied systematically in all EOC hospitals at admission, as well as subsequently potentially at the other hospital interfaces (during transfer and at hospital discharge).

**Objective(s):**	The purpose of this study is to evaluate whether obtaining a BPMH and performing medication reconciliation at hospital admission results in improving some specific healthcare outcomes.<br>Primary study objective: The study seeks primarily to determine if obtaining a BPMH and performing medication reconciliation, in comparison with the standard medication history, reduces the proportion of patients with one unplanned all-cause hospital visit (readmission and emergency department visit within 30 days after initial discharge).<br>Secondary study objectives: To assess if BPMH with medication reconciliation, in comparison with the standard medication history, reduces the incidence of adverse drug reactions (ADRs) during hospital stay, shortens length of stay, leads to a reduction in the use of hospital resources, and/or is associated with a decreased number of deaths.
**Outcome(s):**	Primary study outcome:<br>Composite post-discharge healthcare use variable quantified as the proportion of patients with one unplanned all-cause hospital visit (readmission and emergency department visit within 30 days after initial discharge).<br>Secondary study outcomes:<br>•Incidence of ADRs during hospital stay<br>•Length of hospital stay<br>•Number of deaths during hospital stay<br>•Number of resources used during hospital stay (laboratory tests, radiologic exams, echocardiography, electrocardiograms)
**Study design:**	Interventional<br>Allocation: centralized simple randomization<br>Intervention model: parallel assignment
**Inclusion / Exclusion criteria:**	Patients admitted to the inpatient wards of the two regional hospitals Beata Vergine in Mendrisio and La Carità in Locarno, fulfilling one of the following inclusion criteria, will be eligible for the study:<br>-patients aged ≥ 85 years<br>-patients with > 10 drugs at admission<br>Patients with home care as well as patients from elderly homes, meeting the inclusion criteria, will be factored in the study population.<br>Eligible patients will be included one-time only.<br><br>The presence of any of the following exclusion criteria will lead to patient exclusion:<br>-patients admitted to intensive care unit who do not reach inpatient wards<br>-patients who are planned to stay within inpatient wards for less than 48 hours<br>-patients who have been admitted to any of the EOC hospital wards within the previous 3 months and have been discharged at home

**Measurements and procedures:**	<u>Procedure</u>: Eligible patients, randomized within the intervention arm, will receive medication reconciliation according to the following steps:<br>1) The pharmacy assistant will obtain the BPMH by compiling a comprehensive list of the medications the patient is taking and details about how the drugs are taken. In order to confirm the accuracy of the history, the pharmacy assistant will use at least two sources of information, one of which being, when possible, the interview with the patient and/or family members, in addition to referral letters, prescriptions and drug lists from primary care centres, and other.<br>2) The clinical pharmacist will reconcile BPMH with prescribed medicines and, to resolve unclear or ambiguous discrepancies between the two lists and/or to propose any adaptations of the pharmacotherapy, the clinical pharmacist will refer to the medical doctor.<br>3) The medical doctor will decide potential changes in pharmacotherapy and communicate them to the patient providing complete information on medicines.<br>Medication reconciliation will be applied at hospital admission accordingly to the following time-schedules:<br>A) Patients admitted to the inpatient ward from Monday 7 am to Friday 12 pm: medication reconciliation within 48 hours<br>B) Patients admitted to the inpatient ward from Friday 12 pm to Monday 7 am: medication reconciliation within 48 hours, calculated starting from Monday morning at 7 am<br>C) Patients admitted from the 12 pm of the pre-holiday or during holidays: medication reconciliation within 48 hours calculated from the first non-holiday day at 7 am<br><br><u>Sampling</u>: A centralized simple randomization list to allocate 1:1 included patients in either the intervention or the control groups will be generated by the clinical trial unit (CTU) of the EOC.<br><br>Measurements:<br>• An *ad hoc* database (the case report form, CRF) will be created by the Information and Communication Technology (ICT) unit of the EOC to systematically record outcome measurements, thus enhancing data quality, reducing the amount of missing or incomplete data, inaccuracies and excessive variability.<br>• Pharmacy assistants and clinical pharmacists will have access to the hospital electronic medical record (EMR) system to register and systematically document the medication reconciliation process. For such reason, GECO (Gestione COordinata del paziente), the tool developed and used within the EOC to manage EMRs, has been adapted accordingly by the ICT.<br>• Data quality will be promoted through the training of the pharmacy assistants involved in medication reconciliation: clinical pharmacists-held training sessions are ongoing to inform and explain the specific changes applied in GECO for the registration of the medications' list.<br>• To measure the incidence of ADRs developed by patients during the hospital stay, the active pharmacovigilance system currently in use at the Centro Regionale di Farmacovigilanza del Canton Ticino, will be exploited. EMRs identified through this system and referring to patients from both the two study sites, aged ≥85 years or with more than 10 medications at admission, will be manually validated (to discard false positive cases). Subsequently, ADRs will be categorized as not serious or serious (according to the WHO seriousness criteria, for resulting in death, being life threatening, prolonging hospitalization, resulting in persistent disability or incapacity and other). Outcome assessor will be masked to group allocation. At the end of the analysis, ADRs will be matched to either the intervention or the control group.<br>• Emergency department visits, readmissions, the length of hospital stay and deaths during hospitalization, will be retrieved from administrative data sources (already in use within the EOC for other purposes) with the support of the ICT via an appropriate software able to select the EMRs of interest (referred to included patients).<br>• For the quantitative evaluation of the resources used during

**Study Product / Intervention:**	n.a.
**Control Intervention (if applicable):**	Patients of the control group will not receive BPMH with medication reconciliation at admission. The standard physician-acquired medication history will be performed as usual.
**Number of Participants with Rationale:**	N=1890, considering:<br>• Power 0.80<br>• Significance level 0.05<br>• Expected subsequent hospital visit rate in the control group, 30%<br>• Difference in subsequent hospital visit rates between the control and the intervention group considered clinically relevant, 6%<br>• Patient drop-out, 10%
**Study Duration:**	14 months for the study site La Carità in Locarno, 13 months for the study site Beata Vergine in Mendrisio
**Study Schedule:**	05/11/2018 – 31/01/2020

**Investigator(s):**	Prof. Dr. med. Alessandro Ceschi<br>Medical and scientific director<br>Istituto di Scienze Farmacologiche della Svizzera Italiana<br>Clinica di Farmacologia e Tossicologia Clinica<br>Ente Ospedaliero Cantonale<br>Via Tesserete 46<br>6903 Lugano – Switzerland<br>Email: alessandro.ceschi@eoc.ch<br>Telephone: +41 91 8116863<br><br>Prof. Dr. med. Paolo Ferrari<br>Chief Medical Officer<br>Direzione Generale<br>Ente Ospedaliero Cantonale<br>Viale Officina 3<br>6500 Bellinzona – Switzerland<br>Email: paolo.ferrari@eoc.ch<br>Telephone: +41 91 8111436<br><br>PD Dr. med. Alberto Pagnamenta<br>Head of the Clinical Epidemiology and Biostatistics Unit<br>Area Formazione Accademica, Ricerca e Innovazione<br>Ente Ospedaliero Cantonale<br>Viale Officina 3<br>6500 Bellinzona - Switzerland<br>Email: alberto.pagnamenta@eoc.ch<br>Telephone: +41 91 8113429<br><br>Roberta Noseda, PhD<br>Scientific collaborator<br>Istituto di Scienze Farmacologiche della Svizzera Italiana<br>Clinica di Farmacologia e Tossicologia Clinica<br>Ente Ospedaliero Cantonale<br>Via Tesserete 46<br>6903 Lugano – Switzerland<br>Email: roberta.noseda@eoc.ch<br>Telephone: +41 91 8116300<br><br>Michela Pironi, MSc<br>Clinical pharmacist<br>Istituto di Scienze Farmacologiche della Svizzera Italiana<br>Ente Ospedaliero Cantonale<br>Ospedale Regionale di Locarno<br>Via all'Ospedale 1<br>6600 Locarno – Switzerland<br>Email: michela.pironi@eoc.ch<br>Telephone: +41 91 8114561<br><br>Stefano Bruni, BSc<br>Computer scientist<br>Informatica e Tecnologia della Comunicazione<br>Ente Ospedaliero Cantonale<br>Viale Stefano Franscini 4<br>6500 Bellinzona – Switzerland<br>Email: stefano.bruni@eoc.ch<br>Telephone: +41 91 8111364
**Study Centre(s):**	Regional hospitals Beata Vergine in Mendrisio and La Carità in Locarno

**Statistical Considerations:**	<u>Data Analysis</u>: Quantitative data will be summarized as mean with standard deviation (SD) or as median with interquartile range (IQR) as appropriate. Qualitative data will be presented as absolute numbers with percentages. Comparisons of data between the two populations (patients with medication reconciliation *versus* patients without, corresponding to usual care) will be performed with the Student t-test, Mann-Whitney test, chi-squared test, or Fisher exact test, as appropriated. Primary endpoint is a composite post-discharge healthcare use variable quantified as the proportion of patients with one unplanned all-cause hospital visit (readmission and emergency department visit within 30 days after initial discharge). Subsequent hospital visit rates will be compared between groups with the chi-squared test or the Fisher exact test as appropriate. All tests will be two-sided and p-value < 0.05 will be considered to be statistically significant. Moreover, we will perform a time to event analysis with the Kaplan-Meier method. In this analysis the outcome of interest will be the previously described composite post-discharge healthcare use variable. Time to event curves of patients with and without medication reconciliation will be presented, and thereafter compared with the log-rank test. Unadjusted and adjusted hazard ratios with the corresponding 95% confidence intervals (CI) will be calculated using a Cox proportional hazard model. The proportional hazard assumption will be verified graphically and with the Schoenfeld test. If a variable will violate the proportional hazard assumption, this variable will be used to stratify the Cox model. All statistical analyses will be performed with Stata version 15 (StataCorp LP, College Station, TX, USA).<br><br><u>Sample size assessment</u>: Based on previous studies (**20**) and on the EOC electronic database, we will expect to observe a positive composite variable in about 30% of patients in the control group. To detect a clinically relevant difference (set at 6%) in this composite variable between intervention and control group, with a type I error of 0.05 and a power of 0.80, we determined a sample size of 1718 patients. Taking into account a possible drop-out for different reasons of about 10%, we calculated the definitive sample size of 1890 patients. Sample size calculation was performed with PASS version 15.0.3 (NCSS, LCC, Kaysville, Utah, USA).
**GCP Statement:**	This study will be conducted in compliance with the protocol, the current version of the Declaration of Helsinki, the ICH-GCP or ISO EN 14155 (as far as applicable) as well as all national legal and regulatory requirements.

ABBREVIATIONS

BASEC	Business Administration System for Ethical Committees, (https://submissions.swissethics.ch/en/)
CEC	Competent Ethics Committee
CRF	Case Report Form
ClinO	Ordinance on Clinical Trials in Human Research *(in German: KlinV, in French: OClin, in Italian: OSRUm)*
GCP	Good Clinical Practice
Ho	Null hypothesis
H1	Alternative hypothesis
HRA	Federal Act on Research involving Human Beings *(in German: HFG, in French: LRH, in Italian: LRUm)*
IIT	Investigator-initiated Trial
ISO	International Organisation for Standardisation
ITT	Intention to treat
ICH	International Conference on Harmonization
PI	Principal Investigator
SDV	Source Data Verification
SOP	Standard Operating Procedure
TMF	Trial Master File
WHO	World Health Organization
ICTRP	International Clinical Trial Registry Portal Platform
SNCTP	Swiss National Clinical Trial Portal
EOC	Ente Ospedaliero Cantonale
ISFSI	Istituto di Scienze Farmacologiche della Svizzera Italiana
ADE	Adverse Drug Event
BPMH	Best Possible Medication History
ICT	Information and Communication Technology
CTU	Clinical Trial Unit
EMR	Electronic Medical Record
GECO	GEstione Coordinata del paziente
ADR	Adverse Drug Reaction
SD	Standard Deviation
IQR	Interquartile Range

STUDY SCHEDULE

	Enrolment	Allocation	Post-allocation			Close-out	
**TIMEPOINT**	0	0	Within 48 h from admission (sequentially)			30 days	12 months
ENROLMENT:							
Eligibility screen	X						
Allocation		X					
INTERVENTIONS:							
Standard medication history	X						
BPMH*			X				
MedRec**				X			
Patient communication					X		
OUTCOME MEASURES:							
N. of readmissions						X	
N. of ED*** visits						X	
Death (yes/no)							X
Length of hospital stay							X
N. of ADRs****							X
N. of resources used							X
N. of discrepancies							X
MedRec duration							X

*Best Possible Medication History
**Medication Reconciliation
***Emergency Department
****Adverse Drug Reactions

1. STUDY ADMINISTRATIVE STRUCTURE

1.1 Sponsor, Sponsor-Investigator

Sponsor:
Ente Ospedaliero Cantonale
Contact details:
Viale Officina 3
6501 Bellinzona - Switzerland
Email: direzione.generale@eoc.ch
Telephone: +41 91 8111301

1.2 Principal Investigator(s)

Principal Investigator:
Prof. Dr. med. Alessandro Ceschi
Contact details:
Via Tesserete 46
6903 Lugano - Switzerland
Email: alessandro.ceschi@eoc.ch
Telephone: +41 91 8116863

1.3 Statistician ("Biostatistician")

PD Dr. med. Alberto Pagnamenta
Contact details:
Via Turconi 23
6850 Mendrisio - Switzerland
Email: alberto.pagnamenta@eoc.ch
Telephone: +41 91 8113429

1.4 Laboratory

n.a.

1.5 Monitoring institution

The Sponsor will monitor the study

1.6 Data Safety Monitoring Committee

n.a.

1.7 Any other relevant Committee, Person, Organisation, Institution

Prof. Dr. med. Paolo Ferrari, Chief Medical Officer of the EOC
Contact details:
Viale Officina 3
6500 Bellinzona – Switzerland
Email: paolo.ferrari@eoc.ch
Telephone: +41 91 8111436

Roberta Noseda, PhD, scientific collaborator
Contact details:
Via Tesserete 46
6903 Lugano – Switzerland
Email: roberta.noseda@eoc.ch
Telephone: +41 91 8116300

Michela Pironi, MSc, clinical pharmacist
Contact details:
Via all’Ospedale 1
6600 Locarno – Switzerland
Email: michela.pironi@eoc.ch
Telephone: +41 91 8114561

Stefano Bruni, BSc, computer scientist
Contact details:
Viale Stefano Franscini 4
6500 Bellinzona – Switzerland
Email: stefano.bruni@eoc.ch
Telephone: +41 91 8111364

2. ETHICAL AND REGULATORY ASPECTS

The decision of the CEC concerning the conduct of the study will be made in writing to the Sponsor and the Principal Investigator before commencement of this study. The clinical study can only begin once approval from authorities has been received. Any additional requirements imposed by the authorities shall be implemented.

2.1 Study registration

We intend to register the study at *clinicaltrials.gov* and at *kofam.ch*
The study has been registered at the SNCTP via BASEC.

2.2 Categorisation of study

Study category A: low/absent potential risk for patients exposed to the intervention.
Information concerning patient medication history, gathered during medication reconciliation, will not imply any practical change on patients. This information will be used to correct potential omissions or other types of errors regarding the medication history originally obtained at admission. Medical doctors, not involved in the present study (to whom clinical pharmacists will communicate any type of discrepancy highlighted by medication reconciliation), will be in charge of deciding any change in drug therapy, directly interface with the patients and let them know about any changes in their pharmacotherapy.

2.3 Competent Ethics Committee (CEC)

PD Dr. med. Alessandro Ceschi ensures that approval from the CEC is sought for the clinical study.
No changes will be made to the protocol without prior Principal Investigator and CEC approval.

2.4 Competent Authorities (CA)

n.a.

2.5 Ethical Conduct of the Study

The study will be carried out in accordance to the protocol and with principles enunciated in the current version of the Declaration of Helsinki, the guidelines of Good Clinical Practice (GCP) issued by ICH, and the Swiss Law. The CEC will receive the final report and be informed about study stop/end in agreement with local requirements.

2.6 Declaration of interest

There are no conflict of interest to declare.

2.7 Patient Information and Informed Consent

The investigators will explain to each participant randomized within the intervention arm the nature of the study, its purpose, the procedures involved, the expected duration, the potential risks and benefits and any discomfort it may entail. These participants will be informed that the participation in the study is voluntary and that he/she may withdraw from the study at any time and that withdrawal of consent will not affect his/her subsequent medical assistance and treatment.
The participant must be informed that his/her medical records may be examined by authorised individuals other than their treating physician.
All participants randomized within the intervention arm will be provided a participant information sheet and a consent form describing the study and providing sufficient information for participant to make an informed decision about their participation in the study.
The participant should read and consider the statement before signing and dating the informed consent form, and should be given a copy of the signed document. The consent form must also be signed and dated by the investigator (or his designee) at the same time as the participant sign, and it will be retained as part of the study records.
We ask for exemption from obtaining informed consent from patients randomized within the control arm, for the following reasons:
- obtaining informed consent would introduce a substantial methodological bias with decreased study results' validity;
- all data obtained from the control group are already recorded within the EOC for other, mainly administrative, purposes and no extra data will be obtained only for study purposes;

- as the present study received an internal EOC funding only for increasing the percentage of employment of pharmacy assistants who will obtain the BPMH, obtaining informed consent from patients randomized to the control group would imply significant organizational difficulties due to the lack of available resources;
Instead of performing a simple randomized controlled trial (RCT) (randomization at patient level), a cluster randomized trial (CRT) (randomization at hospital level) would be an interesting alternative design. However CRT is affected by the following technical and ethical limitations:
- CRT usually needs a larger sample size than RCT: data in the same hospital are not independent (correlated). A larger sample size needs more human resources and costs to perform the study;
- to take into account the hierarchical nature of the data permitting to perform a cluster analysis (the reference standard analysis in this situation) a minimum number of cluster is required, and with only four clusters (hospitals) in our study this minimum is not reached;
- the ethical issue in the control group with RCT will remain the same with CRT, because we will gather data from patients in the control hospitals without their formal consent.
A further option could involve emergency physicians obtaining informed consent from patients fulfilling the inclusion criteria before randomization. However, this approach is not feasible as it comes along with several clinical and organizational critical issues:
- it would cause a delay in the emergency physician work, which is challenging and largely unpredictable, since it is a rather time-consuming procedure, which requires that patient has sufficient time to make his/her own decision;
- it would imply that all emergency physicians, as well as ward physicians, should know in detail the study procedures and should be adequately educated on the medication reconciliation process, even if they would otherwise not be involved in the study.

2.8 Participant privacy and confidentiality

Participants’ right to privacy will be upheld by the Sponsor and anonymity of the participants will be guaranteed when presenting the data at scientific meetings or publishing them in scientific journals.
Individual subject medical information obtained as a result of this study is considered confidential and disclosure to third parties is prohibited. Subject confidentiality will be further ensured by utilising subject identification code numbers in computer files.
The CEC has the authority to require direct access to parts of the medical records relevant to the study, including participants’ medical history, for data verification purposes.

2.9 Early termination of the study

The Sponsor or the Principal Investigator may terminate the study prematurely for failure to meet expected enrolment goals and any other administrative reason.

2.10 Protocol amendments

The Principal Investigator is the only person who is allowed to amend the protocol. All amendments will be documented, dated and signed by the Principal Investigator.
Substantial amendments will only be implemented after approval of the CEC.
Under emergency circumstances, deviations from the protocol to protect the rights, safety and well-being of human subjects may proceed without prior approval of the CEC. Such deviations will be documented and reported to the CEC as soon as possible.

3. BACKGROUND AND RATIONALE

3.1 Background and Rationale

Background: Medication reconciliation is a systematic process by which health care professionals obtain the most complete and accurate information about the drugs and other products regularly taken by patients. Aiming at reducing, and possibly avoiding, pharmacological errors (e.g. omissions, additions, dosing errors, drug interactions), medication reconciliation should be performed at all hospital interfaces, especially when new drugs are prescribed or pharmacological therapies are transcribed (**14**).

Internationally, the value of this procedure is mainly attributed to the reduction in the number of adverse drug events (ADEs), which can cause drug-related morbidity and mortality, as well as unnecessary health care costs (**15-17**). It is opinion of the WHO that most of ADEs happen due to a suboptimal communication between healthcare providers and patients at hospital interfaces (admission, transfer, discharge). Moreover, the WHO points out that 67% of drug histories contain one or more errors and 30-80% of the patients have a discrepancy between the drugs taken at home and those prescribed in the hospital (**14, 15**).

Rationale: As part of the *Progress! Pilot project Safe Pharmacotherapy at the interface points* (**18**), promoted by the Federal Office of Public Health, coordinated by the Swiss Patients Safety Foundation and held in several Swiss hospitals, medication reconciliation at hospital admission was introduced at the regional hospital Beata Vergine in Mendrisio, from 2014 to 2016. During this pilot project it was shown that medication reconciliation after obtaining the “Best Possible Medication History” (BPMH) by a pharmacist at hospital admission, in comparison with the standard medication history obtained by the physician at admission, reduced the number of clinically relevant drug discrepancies: among 100 patients included in the project, an average of 5.23 discrepancies per patient was highlighted, and 47% of all patients had a clinically relevant discrepancy potentially evolving in a serious ADE (**19**).

Need for a trial: A structured, well-established and practicable procedure of medication reconciliation that improves patient safety assuring a better quality of care at hospital admission might provide evidence that medication reconciliation could be a valuable intervention to be applied systematically in all EOC hospitals at admission, as well as subsequently potentially at the other hospital interfaces (during transfer and at hospital discharge).

3.2 Investigational Product (treatment, device) and Indication

n.a.

3.3 Preclinical Evidence

n.a.

3.4 Clinical Evidence to Date

Up-to-date there are no systematic reviews and meta-analyses specifically assessing the impact of medication reconciliation performed at admission by pharmacy assistants and clinical pharmacists on clinically relevant healthcare outcomes. In literature, the most relevant data presented as systematic review are those published by *Cheema E et al.* (**20**), where pharmacist-led interventions consisted in medication reconciliation, tailored patient counselling, and provision of telephonic advice to patient at post-hospital discharge. Among the randomized controlled trials included in the meta-analysis, pharmacist-led interventions were effective in reducing medication discrepancies, however, they did not lead to a significant reduction in healthcare utilization (termed as utilization of healthcare resources following drug-related emergency department visits or hospital readmissions).

3.5 Dose Rationale / Medical Device: Rationale for the intended purpose in study (pre-market MD)

n.a.

3.6 Explanation for choice of comparator (or placebo)

The comparator chosen for this study is the “no intervention”, meaning that patients allocated in the control group will not receive BPMH and medication reconciliation at admission. For these patients, the standard physician-acquired drug history will be performed as usual.

3.7 Risks / Benefits

Potential risk for patients exposed to medication reconciliation is almost absent.
Once the clinical pharmacist has reviewed in detail the patient's medication list prescribed after obtaining the standard drug history and compared this with the medication list obtained after BPMH, if any discrepancy is detected, it will be discussed with the patient's physician who will finally define the pharmacotherapy and inform the patient if a change is made.

3.8 Justification of choice of study population

Older patients ≥85 years and/or with more than 10 medications taken at home have been identified by our previous pilot study (see above) as at particular risk for medication discrepancies. Furthermore, older age and polypharmacy have also been identified by other studies as risk factors for discrepancies.
A randomized controlled trial performed by *Gillespie U et al.* (**21**), in patients 80 years or older, showed that pharmacist intervention (on admission, to ensure a correct medication list) is more effective than the standard care (without pharmacist involvement in the health care team at the ward level), with a 16% reduction in all visits to the hospital, and a 47% reduction in the visits to the emergency department, at 12 months follow-up.

4. STUDY OBJECTIVES

4.1 Overall Objective

The purpose of this study is to evaluate whether obtaining a BPMH and performing medication reconciliation at hospital admission results in improving some specific clinically relevant healthcare outcomes.

4.2 Primary Objective

The study seeks primarily to determine if obtaining a BPMH and performing medication reconciliation, in comparison with the standard medication history, reduces the proportion of patients with subsequent unplanned all-cause hospital visits (readmissions and emergency department visits within 30 days after initial discharge).

4.3 Secondary Objectives

To assess if medication reconciliation, in comparison with the standard medication history, reduces the incidence of adverse drug reactions (ADRs) during hospital stay, shortens length of stay, leads to a reduction in the use of hospital resources, and/or is associated to a decreased number of deaths.

4.4 Safety Objectives

n.a.

5. STUDY OUTCOMES

5.1 Primary Outcome

The primary study outcome will be the composite post-discharge healthcare use variable quantified as the proportion of patients with unplanned all-cause hospital visits (including readmissions and emergency department visits within 30 days after initial discharge).
These data will be retrieved from administrative data sources (already in use within the EOC for other purposes) with the support of the ICT via an appropriate software able to select the electronic medical records (EMRs) of interest (referred to included patients).

5.2 Secondary Outcomes

Secondary study outcomes will be: 1) incidence of ADRs during hospital stay; 2) length of hospital stay; 3) number of patients died during hospital stay; 4) number of resources used during hospital stay (laboratory tests, radiologic exams, echocardiography, and electrocardiograms). All these data will be measured at the study close-out (12 months).
To measure the incidence of ADRs developed by patients during the hospital stay, the active pharmacovigilance system currently in use at the Centro Regionale di Farmacovigilanza del Canton Ticino, will be exploited. EMRs identified through this system and referring to patients from both the two study sites, aged ≥85 years or with more than 10 medications at admission, will be manually validated (to discard false positive cases). Subsequently, ADRs will be categorized as not serious or serious (according to the WHO seriousness criteria, for resulting in death, being life threatening, prolonging hospitalization, resulting in persistent disability or incapacity and other). Outcome assessor will be masked to group allocation. At the end of the analysis, ADRs will be matched to either the intervention or the control group.
Length of hospital stay and number of deaths during hospitalization will be retrieved from administrative data sources (already in use within the EOC for other purposes) with the support of the ICT.
For the quantitative evaluation of the resources used during hospitalization, the database already existing for the *Choosing Wisely EOC* project will be used for the counting of laboratory tests, whereas specific softwares will be applied to extrapolate information routinely registered by the EOC for administrative reasons on radiographic exams as well as echocardiography and electrocardiograms.

5.3 Other Outcomes of Interest

n.a.

5.4 Safety Outcomes

n.a.

6. STUDY DESIGN

6.1 General study design and justification of design

The study design is parallel group, two-arm trial with 1:1 allocation.
The two arms are justified as we have two parallel groups, one for the intervention (MedRec) and the other as the control group. The 1:1 allocation is justified in order to have a comparable number of subjects included in both groups.

6.2 Methods of minimising bias

6.2.1 Randomisation

Simple randomization across the two study sites will be centralized: a unique list of randomization will be generated by the clinical trial unit (CTU) of the EOC with an *ad hoc* software, and patients will be allocated 1:1 in either the intervention or the control group.

6.2.2 Blinding procedures

Open-label trial (pharmacy assistants and clinical pharmacists along with study participants randomized to the intervention group will be aware of the study). For study participants allocated within the control group we are asking for the exemption from obtaining informed consent for the reasons stated above (please refer to section 2.7).
Outcome assessors and data analysts will be blinded.

6.2.3 Other methods of minimising bias

n.a.

6.3 Unblinding Procedures (Code break)

n.a.

7. STUDY POPULATION

7.1 Eligibility criteria

Patients admitted to the inpatient wards of the two regional hospitals Beata Vergine in Mendrisio and La Carità in Locarno, fulfilling one of the following <u>inclusion</u> criteria, will be eligible for the study:
-patients aged ≥ 85 years
-patients with > 10 drugs
Patients with home care as well as patients from elderly homes, meeting the inclusion criteria, will be factored in the study population. Eligible patients will be included one-time only.

The presence of any of the following <u>exclusion</u> criteria will lead to patient exclusion:
-patients admitted to intensive care unit who do not reach inpatient wards
-patients who are planned to stay within inpatient wards for less than 48 hours
-patients who have been admitted to any of the EOC hospital wards within the previous 3 months and have been discharged at home

7.2 Recruitment and screening

Patients' selection will take place continuously, as new patients are admitted consecutively to the two hospitals, through GECO – GEstione Coordinata del paziente - platform, which has been opportunely modified by the ICT. Among randomized patients, identification code numbers and arm of study will be assigned with specific softwares.

7.3 Assignment to study groups

The randomization process will be performed by the CTU of the EOC using a simple allocation technique to allocate patients in either the intervention or the control group.

7.4 Criteria for withdrawal / discontinuation of participants

n.a.

8. STUDY INTERVENTION

8.1 Identity of Investigational Products (treatment / medical device)

8.1.1 Experimental Intervention (treatment / medical device)

Eligible patients, randomized within the intervention arm, will receive medication reconciliation according to the following steps:

1) The pharmacy assistant will obtain the BPMH by compiling a comprehensive list of the current medicines the patient is taking and details about how the drugs are taken. To confirm the accuracy of the history, the pharmacy assistant will use at least two sources of information, one of which being, when possible, the interview with the patient and/or family members, in addition to referral letters, prescriptions and drug lists from primary care centres, and other.
2) The clinical pharmacist will reconcile BPMH with prescribed medicines and, to resolve unclear or difficult to evaluate discrepancies between the two lists and/or to propose any adaptations of the pharmacotherapy, the clinical pharmacist will refer to the medical doctor responsible for the patient.
3) The medical doctor will decide potential changes in pharmacotherapy and communicate them to the patient providing complete information on medicines.

Patients will be actively involved only at the beginning of the process, during the interview performed by the pharmacy assistant.

The overall duration of medication reconciliation has been estimated to be 50 to 60 minutes. The first step described above will last about 45-50 minutes, the second step 5 minutes, and the last step less than 5 minutes.

For patients allocated in the control group, the standard physician-acquired drug history will be performed at admission.

Medication reconciliation will be applied at hospital admission accordingly to the following time-schedules:

A) Patients admitted to the inpatient ward from Monday 7 am to Friday 12 pm: medication reconciliation within 48 hours

B) Patients admitted to the inpatient ward from Friday 12 pm to Monday 7 am: medication reconciliation within 48 hours, calculated starting from Monday morning at 7 am

C) Patients admitted from the 12 pm of the pre-holiday or during holidays: medication reconciliation within 48 hours calculated from the first non-holiday day at 7 am

8.1.2 Control Intervention (standard/routine/comparator treatment / medical device)

Patients of the control group will not receive BPMH and medication reconciliation at admission. The standard physician-acquired drug history will be performed as usual.

8.1.3 Packaging, Labelling and Supply (re-supply)

n.a.

8.1.4 Storage Conditions

n.a.

8.2 Administration of experimental and control interventions

8.2.1 Experimental Intervention

Please refer to 8.1.1.

8.2.2 Control Intervention

Please refer to 8.1.2.

8.3 Dose / Device modifications

n.a.

8.4 Compliance with study intervention

n.a.

8.5 Data Collection and Follow-up for withdrawn participants

n.a.

8.6 Trial specific preventive measures

n.a.

8.7 Concomitant Interventions (treatments)

n.a.

8.8 Study Drug / Medical Device Accountability

n.a.

8.9 Return or Destruction of Study Drug / Medical Device

n.a.

9. STUDY ASSESSMENTS

9.1 Study flow chart(s) / table of study procedures and assessments

	**Enrolment**	**Allocation**	**Post-allocation**			**Close-out**	
**TIMEPOINT**	0	0	Within 48 h from admission (sequentially)			30 days	12 months
ENROLMENT:							
Eligibility screen	X						
Allocation		X					
INTERVENTIONS:							
Standard medication history	X						
BPMH*			X				
MedRec**				X			
Patient communication					X		
OUTCOME MEASURES:							
N. of readmissions						X	
N. of ED*** visits						X	
Death (yes/no)							X
Length of hospital stay							X
N. of ADRs							X
N. of resources used							X
N. of discrepancies							X
MedRec duration							X

*Best Possible Medication History
**Medication Reconciliation
***Emergency Department
****Adverse Drug Reactions

9.2 Assessments of outcomes

9.2.1 Assessment of primary outcome

Already described under 5.1.

9.2.2 Assessment of secondary outcomes

Already described under 5.2.

9.2.3 Assessment of other outcomes of interest

n.a.

9.2.4 Assessment of safety outcomes

n.a.

9.2.4.1 Adverse events

n.a.

9.2.4.2 Laboratory parameters
n.a.

9.2.4.3 Vital signs
n.a.

9.2.5 Assessments in participants who prematurely stop the study
n.a.

9.3 Procedures at each visit
n.a.

9.3.1 Split into subtitles by type of visit
n.a.

9.3.2 Split into subtitles by type of visit
n.a.

9.3.3 Split into subtitles by type of visit
n.a.

10. SAFETY

ADRs developed by patients during the hospital stay will be collected through the active pharmacovigilance system (as described under 5.2). As first, the assessor will draw false positive cases apart, then, he will categorize ADRs as either not serious or serious (according to the WHO seriousness criteria - for resulting in death, being life threatening, prolonging hospitalization, resulting in persistent disability or incapacity, and other). The assessor will be masked to group allocation. Finally, ADRs will be matched to either the intervention or the control group.
Validated ADRs will be reported to the Centro Regionale di Farmacovigilanza del Canton Ticino for causality assessment and notification to Swissmedic.

10.1 Drug studies

n.a.

10.1.1 Definition and assessment of (serious) adverse events and other safety related events

n.a.

10.1.2 Reporting of serious adverse events (SAE) and other safety related events

n.a.

10.1.3 Follow up of (Serious) Adverse Events

n.a.

10.2 Medical Device Category C studies

10.2.1 Definition and Assessment of (Serious) Adverse Events and other safety related events

n.a.

10.2.2 Reporting of (Serious) Adverse Events and other safety related events

n.a.

10.2.3 Follow up of (Serious) Adverse Events

n.a.

10.3 Medical Device Category A studies

10.3.1 Definition and Assessment of safety related events

n.a.

10.3.2 Reporting of Safety related events

n.a.

10.4 Assessment, notification and reporting on the use of radiation sources

n.a.

11. STATISTICAL METHODS

11.1 Hypothesis

Null hypothesis → H0: P1 - P2 = 0
Alternative hypothesis → H1: P1 - P2 = D1 ≠ 0

P1 is the proportion of patients of the control group with post-discharge healthcare use, set at 0.30.

P2 is the proportion of patients of the intervention group with post-discharge healthcare use, defined as D1 (difference P1 - P2), and set at 6%

Significance level of 0.05, power of 0.80

Statistic test used: Pearson’s chi-square test

11.2 Determination of Sample Size

Based on previous studies (**20**) and on data extracted from the EOC electronic database, we expect to observe a positive composite variable in about 30% of the patients in the control group. To detect a clinically relevant difference (set at 6%) in this composite variable between intervention and control groups, a sample size of 1718 patients has been calculated. Taking into account a possible drop-out for different reasons of about 10%, we calculated the definitive sample size of 1890 patients. Sample size calculations have been performed with PASS version 15.0.3 (NCSS, LCC, Kaysville, Utah, USA).

11.3 Statistical criteria of termination of trial

Termination of trial is not expected.

11.4 Planned Analyses

11.4.1 Datasets to be analysed, analysis populations

Quantitative data will be summarized as mean with standard deviation (SD) or as median with interquartile range (IQR) as appropriate. Qualitative data will be presented as absolute numbers with percentages. Comparisons of data between the two populations (patients with medication reconciliation *versus* patients without, corresponding to usual care) will be performed with the Student t-test, Mann-Whitney test, chi-squared test, or Fisher exact test, as appropriated. Primary endpoint is a composite post-discharge healthcare use variable quantified as the proportion of patients with one unplanned all-cause hospital visit (readmission and emergency department visit within 30 days after initial discharge). Subsequent hospital visit rates will be compared between groups with the chi-squared test or the Fisher exact test as appropriate. All tests will be two-sided and p-value < 0.05 will be considered to be statistically significant. Moreover, we will perform a time to event analysis with the Kaplan-Meier method. In this analysis the outcome of interest will be the previously described composite post-discharge healthcare use variable. Time to event curves of patients with and without medication reconciliation will be presented, and thereafter compared with the log-rank test. Unadjusted and adjusted hazard ratios with the corresponding 95% confidence intervals (CI) will be calculated using a Cox proportional hazard model. The proportional hazard assumption will be verified graphically and with the Schoenfeld test. If a variable will violate the proportional hazard assumption, this variable will be used to stratify the Cox model. All statistical analyses will be performed with Stata version 15 (StataCorp LP, College Station, TX, USA).

11.4.2 Primary Analysis

Primary analysis will be performed by the biostatistician in order to determine the proportion of patients with one unplanned all-cause hospital visit (including readmission and emergency department visit within 30 days after initial discharge), in both the intervention and the control groups.

11.4.3 Secondary Analyses

Secondary analyses will be performed to measure: 1) the incidence of ADRs during hospital stay; 2) the length of hospital stay; 3) the number of patients died during hospital stay; 4) the number of resources used during hospital stay (laboratory tests, radiologic exams, echocardiography, and electrocardiograms). All these data will be measured at the study close-out (12 months).
To measure the incidence of ADRs developed by patients during the hospital stay, the active pharmacovigilance system currently in use at the Centro Regionale di Farmacovigilanza del Canton Ticino, will be exploited. EMRs identified through this system and referring to patients from both the two study sites, aged ≥85 years or with more than 10 medications at admission, will be manually

validated by the scientific collaborator (to discard false positive cases). Subsequently, ADRs will be categorized as not serious or serious (according to the WHO seriousness criteria, for resulting in death, being life threatening, prolonging hospitalization, resulting in persistent disability or incapacity and other). Outcome assessor (the scientific collaborator) will be masked to group allocation. At the end of the analysis, ADRs will be matched to either the intervention or the control group.
Length of hospital stay and the number of deaths during hospitalization will be retrieved by the ICT from administrative data sources (already in use within the EOC for other purposes).
For the quantitative evaluation of the resources used during hospitalization, the database already existing for the *Choosing Wisely EOC* project will be exploited by the ICT for the counting of laboratory tests, whereas specific softwares will be applied to extrapolate information routinely registered by the EOC for administrative reasons on radiographic exams as well as echocardiography and electrocardiograms.
A subgroup analysis will be performed for patients with a medication dispenser delivered by pharmacy.

11.4.4 Interim analyses

n.a.

11.4.5 Safety analysis

n.a.

11.4.6 Deviation(s) from the original statistical plan

n.a.

11.5 Handling of missing data and drop-outs

Missing data and drop-outs will not be replaced.

12. QUALITY ASSURANCE AND CONTROL

12.1 Data handling and record keeping / archiving

In order to compile the BPMH, the pharmacy assistant will have to consult at least two sources of information. The most important source will be patient (and/or familiars) interview. Documents I-EOFARM-001, P-EOFARM-010 and I-EOFARM-159 have been prepared to guide and support this activity. In order to retrieve information on patients' pharmacotherapy before hospital admission from external pharmacies and general practitioners, provided that the patient gives his/her consent, the document M-EOFARM-050 has been predisposed. The interview will encompass questions about adherence to and understanding of drug therapy regimen, problems and adverse effects, and use of over-the-counter drugs, complementary and alternative medicines. If necessary, the pharmacy assistant will contact territorial health care facilities (family doctor office, pharmacy).

- I-EOFARM-001: "Linee guida per l'intervista per la rilevazione della migliore anamnesi farmacologica possibile"
- P-EOFARM-010: "Studio riconciliazione farmacologica EOC: il processo di verifica sistematica della farmacoterapia all'ammissione"
- I-EOFARM-159: "Migliore anamnesi farmacologica possibile: punti chiave per l'intervista al paziente"
- M-EOFARM-050: "Richiesta di trasmissione delle informazioni riguardanti la terapia farmacologica pre-ammissione"

Throughout the study period, regular meetings of the multi-professional group of investigators will be conducted to assess the study progress.

Eligible patients will be identified through GECO, which has been opportunely adapted for the study, to select patients meeting the inclusion criteria. The CTU will provide the randomization list which will be used to allocate eligible patients within one of the two arms of the study. All patients' related variables, along with identification code numbers and allocation groups, will be gathered within GECO and subsequently transferred to the Case Report Form (CRF). In comparison with paper-based data collection, the use of electronic handheld devices has the potential to improve protocol adherence, data accuracy, user acceptability, and timeliness of receiving data.

Local study pharmacy assistants have been trained to learn about medication reconciliation procedure and to get practice with the use of the modified version of GECO, thus potentially enhancing data quality, reducing the amount of missing or incomplete data, inaccuracies, and excessive variability in measurements.

12.1.1 Case Report Forms

Data concerning study patients will be recorded within an electronic CRF.
Participants will be identified with a numerical coding system (defined by the ICT), and patients' names, initials, and birth dates, will not be reported. Age will be expressed as years at hospital admission.
The ICT (responsible for data management), and the biostatistician, will have the authorization for data entry in the CRF.

12.1.2 Specification of source documents

Among data recorded on the CRF, patient age at admission, patient sex, number of drugs at admission, number of medication discrepancies, number of unjustified drugs added (committed drugs), number of omitted drugs, number of incorrect drug frequency, number of incorrect drug name, number of incorrect dose strength, number of incorrect drug formulations, interview duration, BPMH duration and overall medication reconciliation procedure duration (BPMH + medication reconciliation), will be extrapolated from GECO (where the clinical pharmacist register this information).

Administrative data registries (used within the EOC for other purposes), will be the data source for the number of all-cause hospital visits (readmissions and emergency department visits), the length of hospital stay, and the occurrence of death events, involving study participants. This information will be integrated by the ICT within the CRF.

For the quantitative evaluation of the resources used during hospitalization, the database already existing for the *Choosing Wisely EOC* project will be used for the counting of laboratory tests, whereas specific softwares will be applied to extrapolate information routinely registered by the EOC for administrative reasons on radiographic exams as well as echocardiography and electrocardiograms. All information concerning study participants will be integrated by the ICT within the CRF.

12.1.3 Record keeping / archiving

All study data will be archived without time restrictions after study termination.

12.2 Data management

12.2.1 Data Management System

Data will be managed at first in GECO, where pharmacy assistants and clinical pharmacists will enter patient pharmacotherapy information. Subsequently, identification code numbers and allocation groups will be assigned to included patients. Data will be transferred from GECO to the CRF. Additional outcome measures, extracted from administrative data sources, will be implemented in the CRF by the ICT. For the assessment of ADRs' incidence, the outcome assessor will use GECO to retrieve EMRs and validate potential ADRs. Subsequently these data will also be transferred to the CRF.

A pilot testing of the data management system will be performed one month before the start of the study to assess its reliability and validity.

12.2.2 Data security, access and back-up

Access to GECO and CRF will be protected, only authorized people will be able to access data. Accesses will be tracked in time, modifications and correspondent authors registered.

Backup systems have been predisposed by the ICT.

12.2.3 Analysis and archiving

Data will be extracted from the source via scheduled ETL Job and stored in EOC's central Datawarehouse, where data will be cleaned, normalized and then published in a datamart. Access to datamart will be restricted to eligible users. The process will run every night and data will be maintained depending on the study's needs.

12.2.4 Electronic and central data validation

For validating data from the source, an approach based on the validation of internal consistency concepts, which uses expected associations within the dataset itself (e.g. completeness, uniformity and plausibility), will be applied.

12.3 Monitoring

The Principal Investigator will constantly monitor the source data and all study related documents.

12.4 Audits and Inspections

Source data and all study related documents will be accessible to auditors/inspectors of the CEC.

12.5 Confidentiality, Data Protection

Direct access to source documents will be permitted for purposes of monitoring (12.3), audits and inspections (12.4).

During the study:

- The Sponsor and the Principal Investigator will have access to the protocol
- The Principal Investigator, ICT, and the biostatistician, will have access to the dataset (CRF)
- Pharmacy assistants, clinical pharmacists and ICT, will have access to GECO for study purposes
- The scientific collaborator in charge of measuring the incidence of ADRs during hospitalization will have access to patients' EMRs (in GECO), to validate and assess ADR alerts raised by the active pharmacovigilance system.

After the study, the Principal Investigator will have ultimate authority over any publication and dissemination activity.

12.6 Storage of biological material and related health data

n.a.

13. PUBLICATION AND DISSEMINATION POLICY

We plan to communicate trial results to the Direzione Generale of the EOC. Afterwards, healthcare professionals will be informed about the study results and a publication in an international peer-reviewed scientific journal will be actively considered and pursued. To this purpose, eligible authors will have had to participate in each of the following sections: 1) conception and design of the study, collection and assembly of data, or data analysis and interpretation; 2) writing and reviewing the manuscript; 3) final approval of the manuscript.
The Principal Investigator will have the ultimate authority over any of the above reported activities.

14. FUNDING AND SUPPORT

14.1 Funding

Internal EOC funding for increasing the percentage of employment of pharmacy assistants who will obtain the BPMH. No other funding.

14.2 Other Support

n.a.

15. INSURANCE

n.a.

16. REFERENCES

1. Declaration of Helsinki, Version October 2013, (http://www.wma.net/en/30publications/10policies/b3/index.html)
2. International Conference on Harmonization (ICH, 1996) E6 Guideline for Good Clinical Practice. (http://www.ich.org/fileadmin/Public_Web_Site/ICH_Products/Guidelines/Efficacy/E6/E6_R2__Step_4.pdf)
3. International Conference on Harmonization (ICH, 1997) E8 Guideline: General Considerations for Clinical Trials http://www.ich.org/fileadmin/Public_Web_Site/ICH_Products/Guidelines/Efficacy/E8/Step4/E8_Guideline.pdf)
4. Humanforschungsgesetz, HFG Bundesgesetz über die Forschung am Menschen (Bundesgesetz über die Forschung am Menschen, HFG) vom 30. September 2011/ Loi fédérale relative à la recherche sur l'être humain (loi relative à la recherche sur l'être humain, LRH) du 30 septembre 2011 / Legge federale concernente la ricerca sull'essere umano (Legge sulla ricerca umana, LRUm) del 30 settembre 2011
5. Verordnung über klinische Versuche in der Humanforschung (Verordnung über klinische Versuche, KlinV) vom 20. September 2013 / Ordonnance sur les essais cliniques dans le cadre de la recherche sur l'être humain (Ordonnance sur les essais cliniques, OClin) du 20 septembre 2013. Ordinanza sulle sperimentazioni cliniche nella ricerca umana (Ordinanza sulle sperimentazioni cliniche, OSRUm) del 20 settembre 2013
6. Heilmittelgesetz, HMG Bundesgesetz über Arzneimittel und Medizinprodukte (Heilmittelgesetz, HMG) vom 15. Dezember 2000 / Loi fédérale sur les médicaments et les dispositifs médicaux (Loi sur les produits thérapeutiques, LPT) du 15 décembre 2000 / Legge federale sui medicamenti e i dispositivi medici (Legge sugli agenti terapeutici, LATer)
7. ISO 14155:2011 Clinical investigation of medical devices for human subjects -- Good clinical practice (www.iso.org)
8. ISO 10993 Biological evaluation of medical devices (www.iso.org)
9. MEDDEV 2.7/3 revision 3, May 2015
10. Medizinprodukteverordnung (MepV) vom 17. Oktober 2001 / Ordonnance sur les dispositifs médicaux (ODim) du 17 octobre 2001 / Ordinanza relativa ai dispositivi medici (ODmed) del 17 ottobre 2001
11. WHO, International Clinical Trials Registry Platform (ICTRP) (http://www.who.int/ictrp/en/)
12. European regulation on medical devices 2017/745.
13. Strahlenschutzverordnung (StSV) vom 26. April 2017 / Ordonnance sur la radioprotection (ORaP) du 26 avril 2017 / Ordinanza sulla radioprotezione (ORaP) del 26 aprile 2017.
14. The High 5s Project. Standard Operating Protocol. Assuring Medication Accuracy at Transitions in Care: medication reconciliation. 2014. Available from: http://www.who.int/patientsafety/implementation/solutions/high5s/High5_InterimReport.pdf
15. NICE NPSA Guidance: Medicines optimisation: the safe and effective use of medicines to enable the best possible outcomes. 2015. Available from https://www.nice.org.uk/guidance/ng5
16. Mueller SK, Cunningham S, Kripalani S, Schnipper J. Hospital-based Medication Reconciliation Practices: A Systematic Review. *Arch Intern Med* 2012, 172:1057-1069
17. Kwan JL, Sampson LL, Shojania KG. Medication reconciliation during transitions of care as a patient safety strategy: a systematic review. *Ann Intern Med* 2013, 158:397-403
18. Fishman L, Gehring K, Zimmermann C, Bezzola P. Medication Reconciliation in the Acute Care Hospital (Der systematische Medikationsabgleich im Akutspital). Zürich: Stiftung für Patientensicherheit, 2015
19. Giannini O, Rizza N, Pironi M, Parlato S, Waldispühl B, et al. Errors in medication history: implementation of the medication reconciliation process at admission to a general internal medicine ward. *Submitted*.
20. Cheema E, Alhomoud FK, AL-Deen Kinsara AS, Alsiddik J, Barnawi MH, et al. The impact of pharmacist-led medicines reconciliation on healthcare outcomes in secondary care: a systematic review and meta-analysis of randomized controlled trials. PLOS ONE 2018, https://doi.org/10.1371/journal.pone.0193510
21. Gillespie U, Alassaad A, Henrohn D, Garmo H, Hammarlund-Udenaes M, et al. A comprehensive pharmacist intervention to reduce morbidity in patients 80 years or older. A randomized controlled trial. *Arch Intern Med* 2009, 169(9): 894-900

17. APPENDICES

1. I-EOFARM-001
2. P-EOFARM-010
3. P-EOFARM-159
4. M-EOFARM-050
5. CRF
