# Supplementary material for: Effect of Medication Reconciliation at Hospital Admission on 30-Day Returns to Hospital: A Randomized Clinical Trial
Source: JAMA Netw Open. 2021 Sep 16;4(9):e2124672. doi: 10.1001/jamanetworkopen.2021.24672 (PMC8446815; doi:10.1001/jamanetworkopen.2021.24672)
Supplement: Supplement 2. — eMethods [file jamanetwopen-e2124672-s002.pdf]

Supplemental Online Content

Ceschi A, Noseda R, Pironi M, et al. Effect of medication reconciliation at hospital admission on 30-day returns to hospital: a randomized clinical trial. *JAMA Netw Open*. 2021;4(9):e2124672. doi:10.1001/jamanetworkopen.2021.24672

eMethods.

This supplemental material has been provided by the authors to give readers additional information about their work.

© 2021 Ceschi A et al. *JAMA Network Open.*

eMethods

List of predefined words and word combinations used to assess the period prevalence of adverse drug events (ADEs) occurring during the hospital stay (secondary outcome).

Adverse reaction[s]
Drug-induced
Drug allergy
Pharmacological differential diagnosis
Iatrogen differential diagnosis
Intake differential diagnosis
On iatrogenic
Iatrogenic aetiology
Iatrogenic component
Iatrogenic origin
Drug aetiology
Pharmaco-induced aetiology
Pharmacotherapy
Pharmacologic
Drug origin
Pharmacological origin
Adverse event
On anticoagulation
On anticoagulant therapy
On immunosuppression
On immunosuppressive treatment
On immunosuppressive therapy
On steroid therapy
On corticosteroid therapy
On drug reactions

© 2021 Ceschi A et al. *JAMA Network Open.*

On toxicity from
On polypharmacy
On polypharmacotherapy
On overdose
On contrast medium
On opioid treatment
On opioid treatment
Probable medical cause
Probable pharmacological cause
Probable drug-induced cause
Probable medical cause
Probable medicinal origin
Probable pharmacological origin
Probable drug-induced origin
Pharmaco-toxic origin
Pharmacological aetiology
Drug-induced aetiology
Drug-induced dd
Differential diagnosis by drugs
dd overdose
Differential diagnosis overdose
Therapy-induced
Toxic genesis
Pharmacological genesis
Medicinal genesis
DRESS
Drug reaction with eosinophilia and systemic symptoms
SJS

© 2021 Ceschi A et al. *JAMA Network Open.*

Stevens Johnson syndrome
TEN
Toxic necrotic epidermolysis
Toxic epidermal necrolysis
Lyell
AGEP
Acute generalised exanthematous pustulosis
PML
Progressive multifocal leukoencephalopathy
HIT
Heparin-induced thrombocytopenia
Agranulocytosis Novalgin
Agranulocytosis metamizol
Iatrogenic neutropenia
Neutropenia of medical origin
Neutropenia on therapy
Iatrogenic agranulocytosis
Agranulocytosis of medical origin
Agranulocytosis on therapy
Ferinject extravasation
Ferinject allergic reaction
Ferinject allergy
Ferinject hypersensitivity
Ferinject hypersensitivity reactions
Toxidermia
DILI
Medicinal hepatotoxicity
Iatrogenic hepatotoxicity

© 2021 Ceschi A et al. *JAMA Network Open.*

Hepatotoxicity on therapy
Medicinal nephrotoxicity
Iatrogenic Nephrotoxicity
Nephrotoxicity on therapy
On inadequate intake
On incongruous intake
Medical hepatopathy
Medical hepatitis
Drug interaction
On Marcoumar
On Sintrom
On Xarelto
On Pradaxa
On Eliquis
On Lixiana
Cerebral haemorrhage
Cerebral hematoma
Subarachnoid hematoma
Neuroleptic malignant syndrome
Neuroleptic malignant syndrome
Serotonergic syndrome
Medical pancreatitis
Anaphylactic shock on therapy
Contraceptive.*embolism
Contraceptive.*thrombosis
Contraceptive.*TVP
Pill.*embolism
Pill.*thrombosis

© 2021 Ceschi A et al. *JAMA Network Open.*

Pill.*THB<br><br>Gastrointestinal bleeding.*Marcoumar<br><br>Gastrointestinal bleeding.*Synthrom<br><br>Gastrointestinal bleeding.*Xarelto<br><br>SIADH<br><br>On methotrexate<br><br>On methotrexate<br><br>On mtx
