# Supplementary material for: Effect of Medication Reconciliation at Hospital Admission on 30-Day Returns to Hospital: A Randomized Clinical Trial
Source: JAMA Netw Open. 2021 Sep 16;4(9):e2124672. doi: 10.1001/jamanetworkopen.2021.24672 (PMC8446815; doi:10.1001/jamanetworkopen.2021.24672)
Supplement: Supplement 3. — Data Sharing Statement [file jamanetwopen-e2124672-s003.pdf]

Data Sharing Statement

Ceschi A, Noseda R, Pironi M. Effect of medication reconciliation at hospital admission on 30-day returns to hospital: a randomized clinical trial. *JAMA Netw Open*. 2021;4(9):e2124672. doi:10.1001/jamanetworkopen.2021.24672

Data
Data available: No

Additional Information
Explanation for why data not available: The dataset used and analysed during the current study is available from the corresponding author on reasonable request.
